# Saturday Driving Restrictions Fail to Improve Air Quality in Mexico City

**DOI:** 10.1038/srep41652

**Published:** 2017-02-02

**Authors:** Lucas W. Davis

**Affiliations:** 1 Haas School of Business, University of California, Berkeley, USA.; 2 National Bureau of Economic Research, Cambridge, Massachusetts, USA.

## Abstract

Policymakers around the world are turning to license-plate based driving restrictions in an effort to address urban air pollution. The format differs across cities, but most programs restrict driving once or twice a week during weekdays. This paper focuses on Mexico City, home to one of the oldest and best-known driving restriction policies. For almost two decades Mexico City’s driving restrictions applied during weekdays only. This changed recently, however, when the program was expanded to include Saturdays. This paper uses hourly data from pollution monitoring stations to measure the effect of the Saturday expansion on air quality. Overall, there is little evidence that the program expansion improved air quality. Across eight major pollutants, the program expansion had virtually no discernible effect on pollution levels. These disappointing results stand in sharp contrast to estimates made before the expansion which predicted a 15%+ decrease in vehicle emissions on Saturdays. To understand why the program has been less effective than expected, the paper then turns to evidence from subway, bus, and light rail ridership, finding no evidence that the expansion was successful in getting drivers to switch to lower-emitting forms of transportation.

According to some estimates there will be 1.7 billion cars and trucks on the planet by 2030^1^. Private vehicles bring increased mobility, but also create local air pollution and other negative externalities, particularly in urban areas in low- and middle-income countries where much of this vehicle growth is expected to occur. The World Health Organization estimates that air pollution causes 3.7 million deaths annually^2^, and vehicles play a central role because driving emits large amounts of pollution in close proximity to human populations.

There is wide agreement among economists that externalities should be addressed directly using taxes or cap-and-trade programs. Although there has been some recent progress in this direction, the vast majority of vehicle emissions worldwide remain unpriced. Instead, one of the policy alternatives that is gaining attention is license-plate based driving restrictions. The exact format differs across cities, but most driving restrictions prohibit vehicles from driving once or twice a week during weekdays.

Mexico City has one of the oldest and best-known driving restriction policies. The program bans drivers from using their vehicles one weekday per week based on the last digit of the license plate. For example, vehicles with a license plate ending in “5” or “6” cannot be used on Mondays. For almost two decades the restrictions applied during weekdays only, with weekend driving exempt. Then in July 2008 the program was expanded to include Saturdays.

This paper uses hourly data from pollution monitoring stations to measure the effect of the Saturday expansion on air quality. Pollution levels are compared before and after the program expansion with data from previous years used to control for seasonal and meteorological factors. In the preferred specification, the sample is restricted to a relatively narrow time window around the program expansion and a polynomial time-trend is included to control flexibly for potential omitted variables.

Overall, there is little evidence that the program expansion has improved air quality. Across eight major pollutants, the program expansion had virtually no discernible effect on air quality. The estimated impacts are close to zero and in no specification is there a visual downward shift in pollution levels when the program is expanded. These results stand in sharp contrast to *ex ante* estimates which predicted that the Saturday expansion would decrease vehicle emissions by 15% or more^3^.

To understand why the program is less effective than expected, the paper then turns to evidence from a variety of additional sources. While there was optimism that drivers would substitute to public transportation, daily ridership records show no evidence that the program expansion increased subway, bus, or light rail ridership. These results underscore the difficulty of getting drivers to switch to lower-emitting forms of transportation. Public transportation in Mexico City is widely-available and inexpensive, but can also be slow and uncomfortable and by revealed preference most Mexico City residents choose private vehicles when they have that alternative.

Several features of this paper distinguish it from a small existing literature on driving restrictions. Most significantly, this paper is the first to focus on Saturdays. While previous studies focus on weekdays^4^,^5^,^6^,^7^,^8^, weekends are particularly interesting because travel tends to be more discretionary and with potentially more scope for drivers to substitute to lower-emitting forms of transportation like public buses, or even to zero-emissions forms of transportation like bicycles or walking.

This paper is also significant in that it includes data on airborne particulates. Particulate matter is widely regarded to be the most dangerous pollutant for human health, but previous analyses of driving restrictions in Mexico City have been unable to examine particulates because monitoring stations began collecting data on particulates only relatively recently^4^,^5^.

Finally, this paper distinguishes itself from previous studies by incorporating an unusually wide variety of data sources. In addition to the air pollution and public transportation ridership data, the paper incorporates daily data from the Mexico City Zoo, Mexico City Anthropology Museum, and Mexico City International Airport, as well as less frequent data on gasoline consumption and registered vehicles.

## Results

### Air Quality in North America’s Largest City

Mexico City is the largest city in North America and a natural laboratory for studying air pollution. The term “megacity” is typically used for cities with a population of at least 10 million, and with over 20 million inhabitants Mexico City easily fits this definition. The city’s population is spread well beyond the city’s original historic center and today includes not only the 16 delegations of the Federal District, but also 37 municipalities in the State of Mexico and one municipality in the State of Hidalgo.

Mexico City has some of the worst air quality in the Western Hemisphere, with particulate levels (*PM*_10_) that are three to four times higher than New York, Los Angeles, São Paulo, or Buenos Aires. See the Supplementary materials for details. Mexico City’s chronic problems with air pollution are, in part, the result of the city’s unique geography. Mexico City is located in an elevated basin confined on three sides with mountain ridges that inhibit the horizontal movement of pollutants out of the city, particularly during the winter months.

Record levels of ozone and other airborne pollutants led the Mexico City government to introduce driving restrictions in November 1989. Mexico City was one of the first cities to implement license-plate based driving restrictions, and the policy has spurred similar restrictions in Santiago, São Paulo, Bogotá and elsewhere. Table 1 shows that more than 145 million people live in cities with license-plate based driving restrictions^9^,^10^. Most recently, Delhi launched license-plate based driving restrictions on January 1, 2016.

Mexico City’s program, also known as *Hoy No Circula*, bans drivers from using their vehicles one weekday per week based on the last digit of the vehicle’s license plate. For example, vehicles with a license plate ending in “5” or “6” cannot not be used on Mondays. The restrictions are in place weekdays between 5:00am and 10:00pm and affect both residential and commercial vehicles. When first imposed in 1989, the restrictions applied to 2.3 million vehicles, or 460,000 vehicles per day.

Compliance with the program is near universal. City police vigorously enforce the restrictions and vehicles violating the ban are easy to spot. Drivers caught violating the rules must pay a hefty fine and their vehicles are impounded for 48 hours. Moreover, while it is sometimes possible to avoid these penalties by paying a bribe, the large police presence in Mexico City means that one may need to pay multiple bribes in order to complete even a short trip. In practice, these costs are high enough that most drivers comply assiduously with the program.

Previous research on Mexico City’s weekday restrictions has found disappointing results. There is no evidence that the program initially improved air quality^4^ and air pollution may have actually *increased* 12 to 24 months after the restrictions were imposed^5^. Instead, drivers use taxis more and buy additional cars so that they can drive every day of the week^4^,^5^,^11^,^12^,^13^.

Mexico City’s driving restrictions were expanded to include Saturdays on July 5th, 2008. The primary rationale for the expansion was air quality with the Mexico City government pointing to a steady recent increase in Saturday air pollution levels. As with the weekday requirements, the Saturday ban is based on the last digit of the vehicle’s license plate. For example, vehicles with a license plate ending in “5” or “6” cannot not be used during the first Saturday of the month. The restriction hours (5:00am to 10:00pm) are the same as for weekdays.

Before expanding the program an analysis was performed to predict how the expansion would improve air quality. The program expansion was predicted to reduce Saturday vehicle emissions of carbon monoxide, nitrogen oxides, and large particulates by 17%, 16%, and 16%, respectively^3^. According to emissions inventories for Mexico City, vehicles are responsible for 99% of carbon monoxide, and 82% of nitrogen oxides, but only 23% of large particulates^14^,^15^. Thus for ambient pollution levels the *ex ante* estimates implied the largest decreases for carbon monoxide and nitrogen oxides.

Although the primary rationale for the program expansion was air quality, the introduction of Saturday driving restrictions might also be expected to reduce traffic congestion. According to some estimates the annual cost of traffic delays in Mexico City exceeds $580 per capita with external costs similar in magnitude to the external costs from local air pollutants^16^. Mexico City does not have continuous traffic monitoring so it is not possible to examine traffic congestion directly.

### Air Quality Impacts

Table 2 reports estimates of the effect of driving restrictions on Saturday pollution levels. Overall, there is little evidence that the program expansion improved air quality. The first panel reports estimates for mean daily air pollution, averaged over all hours of the day and all monitoring stations. The estimates are close to zero for all eight pollutants. The point estimate for carbon monoxide is −2.8% and statistically significant at the 5% level, but all other estimates are either positive or very close to zero. The table also reports estimates from a “stacked” specification that shows that the average impact across pollutants is very close to zero.

The second panel reports estimates for maximum daily pollution levels, constructed by averaging pollution levels across monitoring stations for each hour and then taking the maximum for each day. Examining maximum daily pollution levels is particularly important because there are strong nonlinearities in the relationship between pollution and health. The results are again disappointing. Across pollutants, there is no evidence of a decrease in maximum daily pollution levels. The estimate corresponding to carbon monoxide is negative (−1.5%) but not statistically significant, and none of the other estimates are negative.

**Figure 1:**
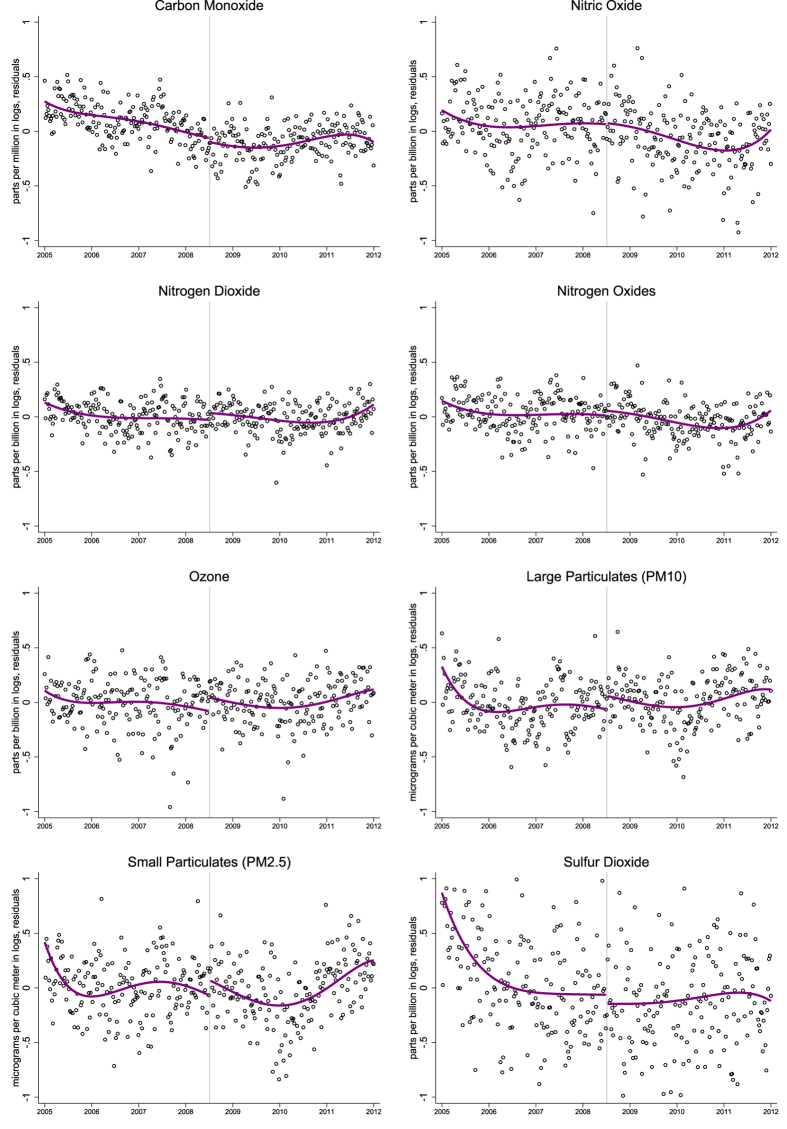
Mean Daily Air Pollution on Saturdays in Mexico City.

Figure 1 provides complementary graphical evidence. These are residual plots using data from Saturdays only between 2005 and 2011. The plots include a fifth-order polynomial and intercept break corresponding to the program expansion. There is no visually discernible decrease in air pollution for any of the eight pollutants. Consistent with the regression estimates, the estimated intercepts are close to zero or positive for most pollutants, and in no case is there a visual downward shift in pollution when the Saturday restrictions begin.

The Supplementary materials include similar plots for maximum pollution levels. Again, there is no visually discernible improvement in air quality when the program is expanded. Estimates are also reported separately by hour-of-the-day. Of 192 total estimates, 107 (56%) are negative so only slightly more than would have been expected due to chance alone. There is a modest decrease for carbon monoxide and nitrogen oxides on Saturday afternoons, but no decrease for any other pollutant during these hours. Moreover, none of the eight pollutants decrease on Saturday mornings. Thus overall there is very little evidence in any specification that the Saturday expansion has reduced air pollution in Mexico City.

### Additional Evidence

The paper now turns to evidence from a variety of additional sources. These ancillary analyses are valuable in helping to understand the air quality results as well as for assessing the extent to which the experience in Mexico City can be generalized to other urban areas.

**Figure 2:**
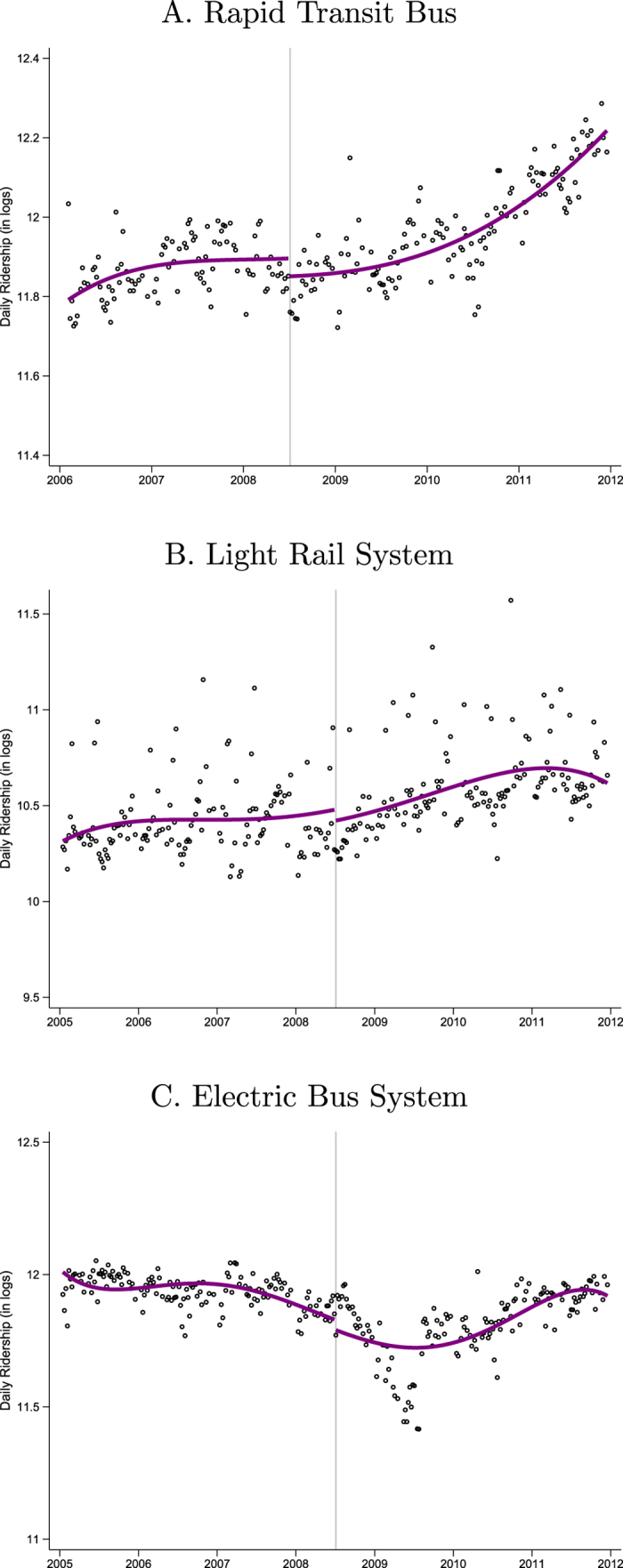
Public Transportation, Saturday Ridership.

It was hoped that the Saturday restrictions would encourage drivers to substitute to public transportation but there has been no attempt to evaluate this empirically. Figure 2 plots daily ridership on Saturdays excluding holidays for three forms of public transportation in Mexico City. Similar to the graphical analyses of the pollution data, these figures include a fifth-order polynomial in time with an intercept corresponding to the program expansion. The Supplementary materials include descriptive statistics and additional analyses including evidence from the Mexico City subway.

There is no evidence that the restrictions induced substitution to any form of public transportation. Rapid transit bus (*Metrobús*) ridership is steadily expanding throughout this period, but there is no discernible increase in ridership when the Saturday restrictions are implemented. Light rail system (*Tren Ligero*) ridership is flat throughout the sample period, with no change at the intercept. Finally, the electric bus system (*Red de Trolebuses*) experiences a sharp decrease then recovery between 2008 and 2009 due to unrelated construction projects but, again, no discernible increase when the Saturday restrictions begin.

This lack of substitution to these lower-emitting forms of transportation is disappointing from a program evaluation perspective but perhaps not surprising. Public transportation in Mexico City is inexpensive but also slower and less convenient than private transportation. Bus and rail transportation follows well-specified major routes, so must typically be combined with other forms of transportation. Public transportation also tends to be crowded, particularly during peak hours when the system runs near capacity. In practice, these disadvantages lead most residents of Mexico City to choose private vehicles when they have that alternative.

**Figure 3:**
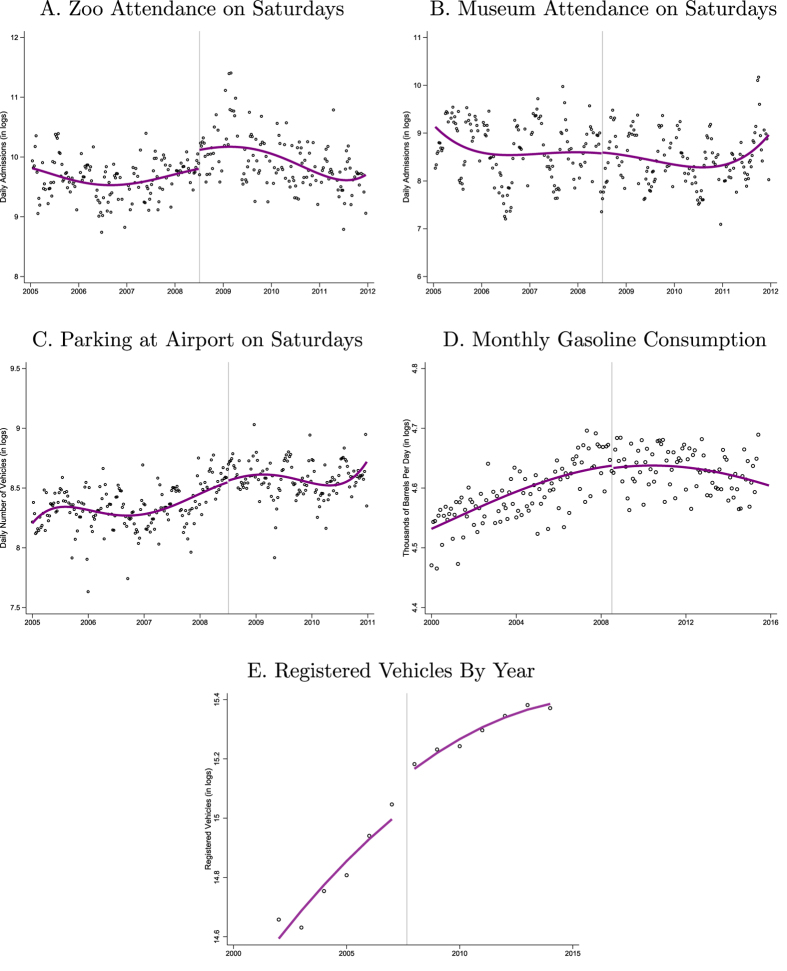
Additional Evidence.

Figure 3 plots data from several additional sources. All outcome variables are again measured in logs and all figures include a flexible polynomial in time with an intercept at July 5th, 2008. If the restrictions were leading drivers to stay home, you would expect to see decreased activity in Mexico City after the program expansion. Moreover, in the medium and long-run you could expect the restrictions to impact gasoline consumption and the number of registered vehicles.

Across measures there is no evidence of a decrease in city activity. Zoo attendance on Saturdays appears to increase, but there is a similar pattern observed on Sundays which suggests that this change is not a result of the program expansion. Attendance on Saturdays at Mexico City’s National Anthropology museum does not decrease nor does the number of cars parked at the Mexico City International airport. Monthly gasoline consumption is flat through July 5th, 2008 and annual data on the number of registered vehicles show a steady increase throughout the 2000 s.

Rather than switching to public transportation or staying at home, the evidence suggests that drivers instead respond to the Saturday restrictions getting rides from other drivers. For households with more than one vehicle, this substitution is natural and relatively easy. For households with only a single vehicle, drivers can ask for a ride from extended family, friends, and neighbors. Whether or not this ride sharing yields a net reduction in driving is not clear. A classic carpool with individuals leaving from and going to the exact same location will reduce total driving, but if drivers are going out of their normal routes then total driving may actually increase.

Taxis are another very relevant alternative. Mexico City has one of the largest taxi fleets in the world. Taxis are available everywhere in the city and are relatively cheap. For a driver who is prohibited from using their vehicle one Saturday per month, taking a taxi is an easy, convenient alternative. Uber was launched in Mexico City in 2013 so was not yet available when the program expanded to Saturdays. Moving forward, however, Uber, Lyft, and similar companies are making taxi-like services even more available.

## Discussion

### Costs Borne by Drivers

Driving restrictions are sometimes incorrectly perceived to be costless because they have little direct impact on the government budgets. But, of course, this ignores the economic cost borne by drivers. Driving restrictions make drivers worse off by preventing them from using a preferred mode of transportation. The magnitude of this welfare loss depends on the next-best mode of transportation and how inferior it is compared to the preferred option.

Willingness-to-pay to avoid the restrictions is not directly observable, but previous studies have attempted to infer it using a couple of different approaches. Many drivers respond to driving restrictions by buying a second vehicle, so vehicle expenditures provide information about willingness-to-pay. This approach requires strong assumptions, but has the advantage of being based on actual decisions made by drivers. Using this approach^4^, finds that driving restrictions impose costs equal to about $3.00 per vehicle per day.

Another point of comparison is taxi fares. Taxis are widely available so waiting times are minimal and the main cost is the fare itself. A 10-kilometer taxi ride with a regular Mexico City taxi costs $3.75 (62 pesos), so drivers who respond to driving restrictions by taking a round-trip taxi ride incur daily costs of $7.50. This back-of-the-envelope calculation misses several important advantages of taxis (avoid parking fees, avoid gasoline and other variable costs), but also several disadvantages (hassle cost, less comfortable, less safe).

Lastly, a recent contingent valuation study asked 2,500 Mexico City drivers how much they would be willing-to-pay to avoid driving restrictions^17^. This is in some ways the most direct approach for quantifying the costs of the program and has the advantage that, at least in theory, these self-reported measures of willingness-to-pay should include both monetary costs (e.g. taxi fares) but also non-monetary costs like loss of time and hassle costs. This study find an average annual willingness-to-pay equivalent to about $2.00 per vehicle per day.

Thus the estimates range from about $2 to $7.50 per day. Under Saturday driving restrictions, most vehicles are banned from driving 12 Saturdays per year, so this is $24 to $90 annually per vehicle. There are about 3.0 million vehicles in Mexico City subject to Saturday driving restrictions, so the total welfare loss from the Saturday expansion ranges from $72 million to $270 million annually.

These costs must be compared to the program’s benefits. The most dangerous pollutants for human health are particulate matter and ozone^18^,^19^. The World Bank finds that the annual benefits from a 10% reduction of PM_10_ for all days of the year in Mexico City would be approximately $920 million, while the annual benefits from a 10% reduction in ozone would be approximately $160 million (both estimates in U.S. 2015 dollars)^20^. Consequently, a 10% reduction of both pollutants on Saturdays would yield benefits of approximately $150 million.

Thus the potential benefits from improved air quality are substantial, and similar in magnitude to the estimated costs. Unfortunately, however, there is no evidence that these benefits are actually realized. Most importantly, there is no evidence that the Saturday expansion decreased airborne particulates or ozone. This lack of evidence of benefits makes it difficult to justify the program in terms of cost-effectiveness regardless of the exact magnitude of the costs borne by drivers.

### Broader Lessons

The world’s population is expected to increase from 7.3 billion today to 8.5 billion by 2030, with most of this growth occurring in urban areas in low- and middle-income countries^21^,^22^. This growth places great environmental stress on the earth’s cities, threatening to exacerbate already severe air pollution problems. Faced with this daunting challenge, it is not surprising that many policymakers are turning to license-plate driving restrictions. Vehicles are a major source of emissions and more visible than many other sources of air pollution.

It is critical, however, for these policies to be evaluated rigorously. The effectiveness of driving restrictions depends on the available substitutes and the willingness of drivers to switch to lower-emissions forms of transportation. Drivers have a revealed preference for fast and convenient transportation so it can be difficult to get them to switch to public transportation. Moving forward, this is not going to get any easier with the increased global availability of taxi-like services like Uber. As incomes continue to increase around the world so does the value of time and thus the preference for private transportation.

## Materials and Methods

The paper also contributes, more broadly, to a growing literature using high-quality data and credible identification techniques to better understand the impact of government policies on air quality^23^,^24^,^25^,^26^. Determining the causal impact of government policies is difficult because one must construct a credible counterfactual for what would have happened to air quality in the absence of the policy. This paper addresses this challenge using a regression discontinuity analysis but there are many quasi-experimental techniques that can be helpful for disentangling causal effects.

This analysis uses hourly air pollution data from the Automated Environmental Monitoring Network (*Red Automático de Monitoreo Atmosférico*), also known as RAMA. This network is one of the most sophisticated air quality monitoring systems anywhere in the world and includes 29 stations distributed throughout Mexico City. Station locations were determined by Mexico City’s Environmental Agency (*Secretaría del Medio Ambiente*) and are intended to reflect a representative sample of neighborhoods in Mexico City. These data have been widely used in previous studies both by economists^4^,^5^,^27^,^28^ and atmospheric scientists^29^,^30^,^31^,^32^.

RAMA tracks hourly measures of carbon monoxide (*CO*), nitric oxide (*NO*), nitrogen dioxide (*NO*_2_), nitrogen oxides (*NO*_*x*_), ozone (*O*_3_), large particulates (*PM*_10_), small particulates (*PM*_2.5_), and sulfur dioxide (*SO*_2_), as well as hourly measures of temperature, humidity, and wind speed. See the Supplementary materials for descriptive statistics and histograms. The stations are located away from direct emission sources and follow norms established by the U.S. Environmental Protection Agency (EPA), including testing of measurement procedures and comparisons against mobile EPA equipment^33^.

Table 2 reports least squares estimates of *γ*_1_ from the following regression equation,


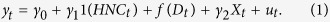


The outcome variable *y*_*t*_ is the pollution level on day *t*, measured in logs. Both mean and maximum daily pollution levels are examined. The explanatory variable of interest is 1(*HNC*), an indicator variable for Saturdays after July 5th 2008. All specifications also include *f*(*D*_*t*_), a fifth-order polynomial in the time (i.e. the “running variable”), and covariates *X*_*t*_ including quartics in temperature, humidity, and wind speed, as well as indicator variables for wind direction, week-of-year and day-of-week. The Supplementary materials include a complete description of the data and empirical strategy as well as estimates from specifications with alternative polynomials and shorter sample periods.

## Additional Information

**How to cite this article**: Davis, L. W. Saturday Driving Restrictions Fail to Improve Air Quality in Mexico City. *Sci. Rep.* **7**, 41652; doi: 10.1038/srep41652 (2017).

**Publisher's note:** Springer Nature remains neutral with regard to jurisdictional claims in published maps and institutional affiliations.

## Supplementary Material

Supplementary Information

## Figures and Tables

**Table 1:** License-Plate Based Driving Restrictions.

City	First Year of Restrictions	Urban Area Population, in millions
Athens, Greece	1982	3.5
Mexico City, Mexico	1989	20.1
Santiago, Chile	1990	6.2
São Paulo, Brazil	1995	20.4
Bogotá, Colombia	1998	9.0
Manila, Philippines	2003	24.1
La Paz, Bolivia	2003	1.9
San Jose, Costa Rica	2005	1.2
Beijing, China	2008	21.0
Tianjin, China	2008	10.9
Quito, Ecuador	2010	1.7
Delhi, India	2016	25.0

**Table 2:** The Effect of Driving Restrictions on Saturday Pollution Levels.

CO	NO	NO_2_	NO_*X*_	O_3_	PM_10_	PM_2.5_	SO_2_	Stacked
A. Mean Pollution, All Hours (in logs)								
−0.028*	−0.010	0.008	−0.001	0.011	0.024	0.027	0.011	0.005
(0.014)	(0.027)	(0.012)	(0.016)	(0.020)	(0.020)	(0.025)	(0.054)	(0.016)
B. Maximum Pollution, All Hours (in logs)								
−0.015	0.013	0.015	0.008	0.000	0.045	0.032	0.026	0.016
(0.022)	(0.035)	(0.019)	(0.024)	(0.024)	(0.026)	(0.031)	(0.073)	(0.019)

Note: This table reports estimates and standard errors from 18 separate regressions all estimated using daily observations from 2005 to 2011. The dependent variable varies across regressions as indicated in the panel and column headings. CO is carbon monoxide, NO is nitric oxides, NO_2_ is nitrogen dioxide, NO_*x*_ is nitrogen oxides, O_3_ is ozone, PM_10_ is large particulates, PM_2.5_ is small particulates, and SO_2_ is sulfur dioxide. All dependent variables are measured in logs and all regressions control for a fifth-order polynomial in time, meteorological variables, and fixed effects for week-of-year and day-of-week. Standard errors, in parentheses, are robust to heteroskedasticity and arbitrary serial correlation within week-of-sample. An asterisk indicates statistical significance at the 5% level.
