# Supplementary material for: Saturday Driving Restrictions Fail to Improve Air Quality in Mexico City
Source: Sci Rep. 2017 Feb 2;7:41652. doi: 10.1038/srep41652 (PMC5289101; doi:10.1038/srep41652)
Supplement: Supplementary Information [file srep41652-s1.pdf]

Saturday Driving Restrictions Fail to Improve Air Quality in Mexico City
Supplementary Information

Lucas W. Davis
University of California, Berkeley

December 2016

1 Air Quality Data

1.1 Histograms

As explained in the paper, the analysis uses hourly air pollution data from the Automated Environmental Monitoring Network (*Red Automático de Monitoreo Atmosférico*), also known as RAMA. Monitoring station locations were determined by Mexico City's Environmental Agency (*Secretaría del Medio Ambiente*) and are intended to reflect a representative sample of neighborhoods in Mexico City.

RAMA tracks hourly measures of carbon monoxide (CO), nitric oxide (NO), nitrogen dioxide (NO_2), nitrogen oxides (NO_x), ozone (O_3), large particulates (PM_{10}), small particulates ($PM_{2.5}$), and sulfur dioxide (SO_2), as well as hourly measures of temperature, humidity, and wind speed. Appendix Table 2 presents descriptive statistics and Appendix Figures 1, 2, and 3 show histograms for all eight pollutants and all four meteorological measures.

1.2 Raw Time Series

Appendix Figure 4 plots average daily pollution levels during the period 2005-2011. This is a 7-year window including approximately 3.5 years before and after the introduction of Saturday driving restrictions. These figures include one observation per monitoring station for each Saturday. Mean daily pollution levels were constructed by averaging over all hours of the day. Overall, pollution levels demonstrate little trend over this period.

For most pollutants there is a pronounced seasonal pattern with higher pollution levels during the winter season due to thermal inversions. In the summer air pollution is less of a problem because the sun warms surface air causing it to rise, carrying pollutants up and out of the city. In the winter, however, the sun provides less warmth and cool surface air is trapped by warmer air above. These temperature inversions cause air quality to be lower during winter months [1, 2, 3].

The vertical line corresponds to July 5th 2008, the day the program was expanded to include Saturdays. There is no discernible decrease corresponding to the program expansion for any of the eight pollutants. Pollution levels are highly variable however, so it is difficult to draw strong conclusions on the basis of these raw data. The analysis in the paper refines this before-and-after comparison by introducing parametric controls for seasonality, weather, and other factors.

1.3 Hourly Pattern During Weekends

Appendix Figures 5 and 6 describe the Friday to Monday pattern for air pollution in Mexico City. These plots were constructed using all observation from 2006 and 2007, the two complete years prior to the introduction of Saturday driving restrictions. Each observation represents mean pollution averaged over all monitoring stations for a specific hour and day during this period. Several days are included in the same plot to facilitate comparison both within and across days.

Carbon monoxide and nitrogen oxides are clearly associated with driving, with peak daily pollution levels reached during the morning commute and then a second, more diffuse peak during the afternoon commute and evening. Carbon monoxide and nitrogen oxides exhibit markedly lower peaks on Saturdays and, in particular, on Sundays. This is consistent with emissions inventories that have been performed for Mexico City indicating that vehicles in Mexico City are responsible for 99% of carbon monoxide and 82% of nitrogen oxides, and 62% of small particulates but only 34% of VOCs and 23% of large particulates [4].

Ozone follows a different pattern, peaking once in the middle of the day when the sun is highest in the sky, and with similar peaks all days of the week. This suggests that ozone production in Mexico City's atmosphere is not limited by ambient nitrogen oxides.[1] That is, ozone levels are relatively insensitive to changes in emissions of nitrogen oxides, and thus are unlikely to have been meaningfully decreased by driving restrictions.

These figures show that air quality responds relatively quickly to changes in vehicle emissions. Exactly how long pollutants remain in the atmosphere depends on wind speed, thermal inversions, and other climatological factors but, overall, the rapid changes over the course of the day indicate that pollutants do not typically remain in the atmosphere for more than a couple of hours. This is important for the analysis that follows because it shows that it should be possible to make inference about changes in emissions by comparing air pollution levels within a narrow time window.

2 Empirical Strategy

Determining the causal impact of driving restrictions is difficult because air quality depends on numerous factors which are constantly evolving. For example, vehicle emissions are constantly changing as households and firms buy new vehicles and change driving patterns, and as the transportation network itself is upgraded and modified. Similarly, industrial emissions are constantly changing as plants open and close, and

[1]Several studies have analyzed ozone production in Mexico City [1, 3]. Ozone is produced by photochemical reactions between nitrogen oxides and volatile organic compounds (VOCs) and the evidence points to ozone production in Mexico City being both VOC-limited and NO_x-inhibited. That is, ozone production increases with VOCs but also *decreases* modestly with NO_x.

as firms adopt new production technologies and emissions control equipment. Consequently, naive before-and-after comparisons will be biased by these other factors that are changing over time.

The approach adopted in this paper is to use a regression discontinuity (RD) analysis. RD is a quasi-experimental research design that exploits the sharpness with which the Saturday driving restrictions were implemented and compares air quality immediately before and after the policy change. The main regression specification used in the analysis is equation (1) in the paper. As described in the paper, all specifications include a fifth-order polynomial in time to control for time-varying omitted variables.

An important advantage of RD is that it requires a considerably weaker identifying assumption than other approaches [5]. Nonparametric identification with RD requires only that the conditional mean function $E[u_t|x_t]$ be continuous at the threshold, t_0. Under this assumption there may be unobserved factors that influence air quality, but their effect cannot change discontinuously at the threshold and the parameter of interest γ_1 is identified within an arbitrarily narrow window around the discontinuity,

$$\gamma_1 = \lim_{t \to t_0^+} E[y|t] - \lim_{t \to t_0^-} E[y|t]. \tag{1}$$

Increasing the interval around this threshold may introduce bias. However, if an assumption is made about the functional form of the relationship between air pollution and time then a wider interval can be used to identify the effect of the policy change. This is why the regressions include the flexible polynomial in time. The RD estimate of γ_1 is unbiased as long as this polynomial accurately captures the time-varying omitted variables.

A limitation of RD is that it only identifies the effect locally at the point in time when the policy change occurred. This is a significant weakness of RD because for policy evaluation one would like to know both the short- and long-run effects of policy changes. Still, the short-run impact is quite informative, particularly in this case. Although the relative size of short- and long-run impacts is theoretically ambiguous [6], in practice the tendency has been for restrictions to increase the vehicle stock, leading to worse air quality in the long-run [7, 8]. If this is indeed the pattern of longer-run adaptations, then finding zero or small short-run impacts is very disappointing news from a policy perspective.

In all specifications the variance matrix is estimated taking into account that u_t may be serially correlated. Standard diagnostic tests were used to assess the magnitude of serial correlation. In the preferred specification, the autocorrelation coefficients are statistically significant only for three or four days. Accordingly, variance matrices are estimated allowing for arbitrary correlation within week-of-sample, defined Thursday through Wednesday. Newey-West standard errors with a seven day lag are also reported as an alternative specification in Appendix Table 3.

2.1 Alternative Specifications

Appendix Figure 7 is identical to Figure 1 in the paper, but uses daily *maximum* pollution levels rather than daily means. As discussed in the paper, the health impacts of air pollution are highly nonlinear, so these results are of large intrinsic interest. Across pollutants, there is no visually discernible improvement in air quality when the program is expanded. Consistent with the regression estimates in Table 2 in the paper, there is no evidence of a downward shift in pollution levels coincident with the beginning of Saturday driving restrictions.

RD estimates can be sensitive to minor changes so it is important to evaluate alternative specifications. Appendix Table 4 reports estimates using alternative polynomials, ranging from third-order to seventh-order. Similarly, Appendix Table 5 reports estimates using shorter windows and less flexible polynomials [9]. As is clarified in the table notes, these specifications are otherwise identical to our baseline estimates.

Overall, the results are very similar with these alternative estimating equations. In many specifications the estimates for carbon monoxide are negative and statistically significant at the 5% level, but estimates for other pollutants are positive or very close to zero across specifications. Moreover, the estimates in the stacked specification are always very close to zero.

Finally, Appendix Table 6 tests for intertemporal substitution toward Fridays and Sundays. There is no evidence that the Saturday driving restrictions increased driving on these other days of the week. The estimate in the stacked specification for Sundays is slightly positive (0.6%) but not statistically significant.

2.2 Impacts by Hour

There are several reasons why the impacts of driving restrictions might differ by hour of the day. First, driving restrictions ban driving only between 5am and 10pm, so one might expect driving to *increase* outside these hours if drivers are able to intertemporally substitute. Second, the composition of drivers and trips may vary across hours, with Saturday afternoon and, in particular, Saturday evening driving including much more discretionary driving than other hours.

Appendix Figure 8 plots RD estimates and 95th percentile confidence intervals for the effect of Saturday driving restriction on air quality by hour-of-the day. Separate regressions are estimated for each pollutant and hour-of-the-day, all with fifth-order polynomials in time, weather variables, and indicator variables for week-of-year and day-of-week. That is, these results are identical to the main regression estimates but estimated hour-by-hour.

Of the 192 total estimates, 107 (56%) are negative, thus only slightly more negative estimates than one would expect due to chance alone. There are negative and sta-

tistically significant estimates for carbon monoxide, nitric oxide, and nitrogen oxides between about 2pm and 10pm, but other pollutants do not have this pattern. Moreover, none of the eight pollutants decrease on Saturday mornings. Thus, overall, the hour-by-hour evidence points to very limited air quality improvements from the Saturday expansion.

2.3 Additional Evidence on Behavioral Responses

These supplementary materials also include plots of raw daily data for the different forms of public transportation and additional measures of economic activity such as Museum attendance. These plots are useful because they show how Saturday activity compares, in general, to the rest of the week, and because they show the overall level and trend in activity over this period. In addition to the measures reported in the paper, these materials include evidence from the Mexico City subway. As can be seen in Appendix Figure 19, there is an increase in subway ridership during all days of the week around July 2008. Because this increase is observed all seven days of the week, this is very unlikely to be related to the Saturday expansion and there is no *differential* increase in ridership on Saturdays relative to the other days of the week.

References

[1] Stephens S, Madronich S, Wu F, Olson JB, Ramos R, Retama A, Muñoz R. Weekly Patterns of Mexico City's Surface Concentrations of CO, NO_x, PM_{10} and O_3 During 1986–2007. *Atmospheric Chemistry and Physics* **8:17**, 5313–5325 (2008).

[2] Parrish DD, Singh HB, Molina L, Madronich S. Air Quality Progress in North American Megacities: A Review. *Atmospheric Environment* **45:39**, 7015–7025 (2011).

[3] Retama, A, Baumgardner D, Raga GB, McMeeking GR, Walker JW. Seasonal and Diurnal Trends in Black Carbon Properties and Co-Pollutants in Mexico City.' *Atmospheric Chemistry and Physics* **15:16**, 9693–9709 (2015).

[4] Molina LT, Madronich S, Gaffney JS, Apel E, de Foy B, Fast J, et al. An Overview of the MILAGRO 2006 Campaign: Mexico City Emissions and their Transport and Transformation. *Atmospheric Chemistry and Physics* **10:18**, 8697–8760 (2010).

[5] Hahn J, Todd P, Van der Klaauw W. Identification and Estimation of Treatment Effects with a Regression-Discontinuity Design. *Econometrica* **69:1**, 201–209 (2001).

[6] Eskeland G, Feyzioglu T. Rationing Can Backfire: The Day Without a Car in Mexico City. *World Bank Economic Review* **11:3**, 383–408 (1997).

[7] Gallego F, Montero J, Salas C. The Effect of Transport Policies on Car Use: A Bundling Model with Applications. *Energy Economics* **40**, S85–S97 (2013).

[8] Gallego F, Montero J, Salas C. The Effect of Transport Policies on Car Use: Evidence from Latin American Cities. *Journal of Public Economics* **107**, 47–62 (2013).

[9] Gelman A, Imbens G. Why High-order Polynomials Should Not Be Used in Regression Discontinuity Designs. *National Bureau of Economic Research Working Paper* (2014).

Appendix Figure 1: Air Pollution, Histograms

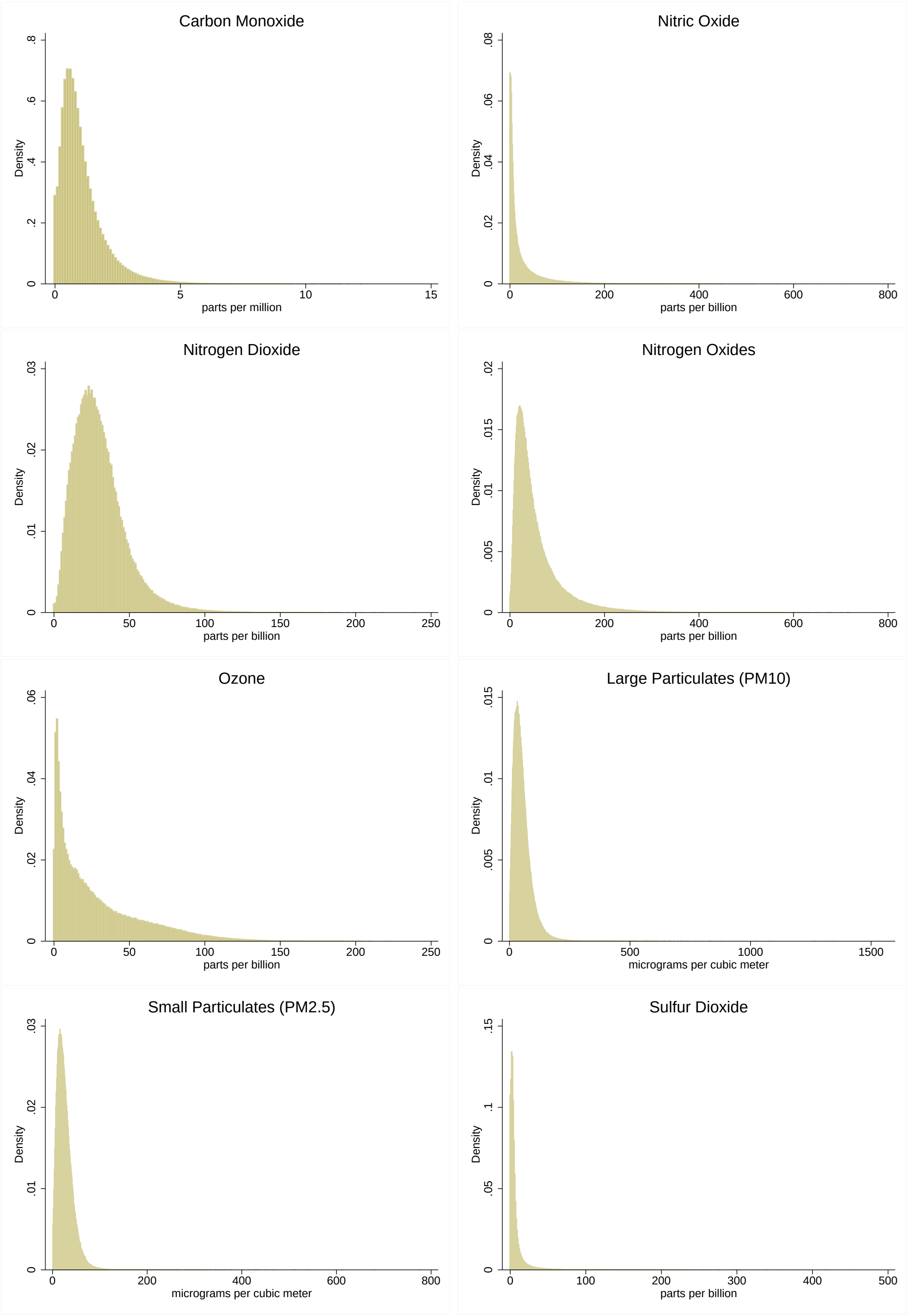

Appendix Figure 2: Air Pollution, Truncated Histograms with Top 1% Dropped

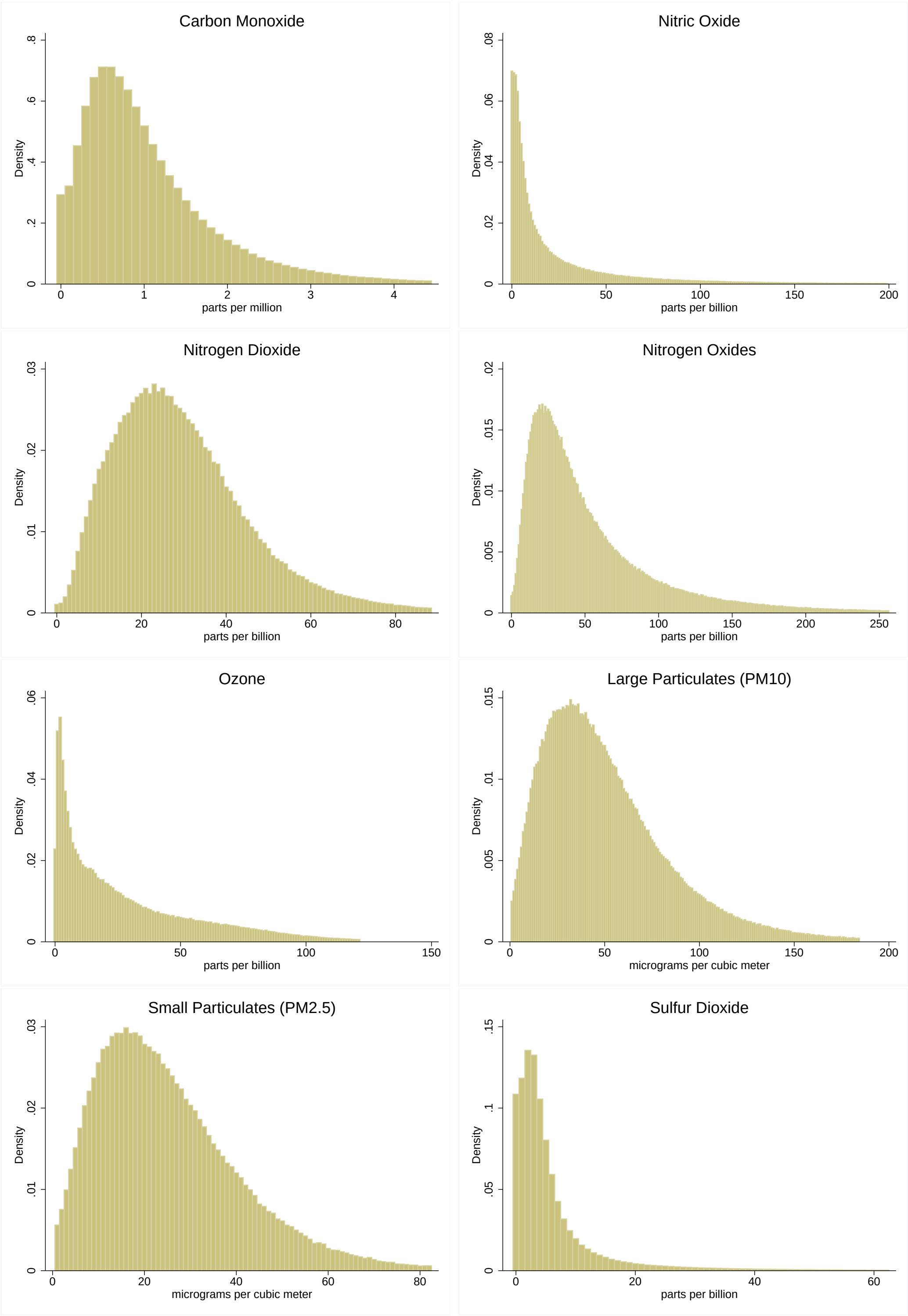

Appendix Figure 3: Weather Variables, Histograms

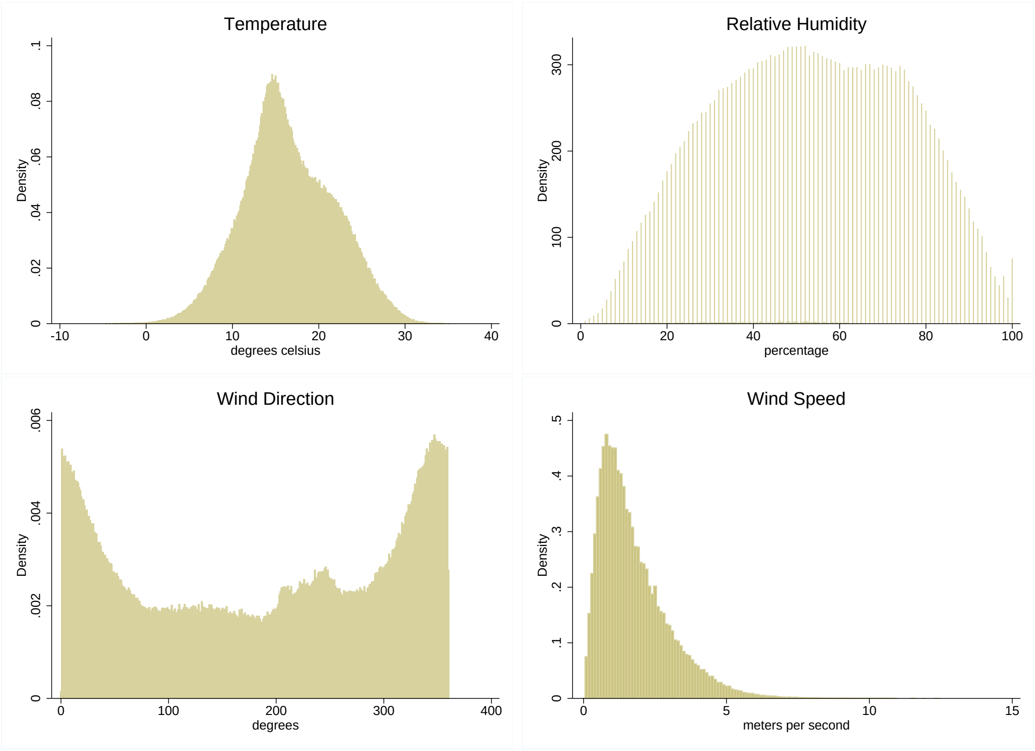

Appendix Figure 4: Mean Daily Air Pollution on Saturdays in Mexico City 2005-2011

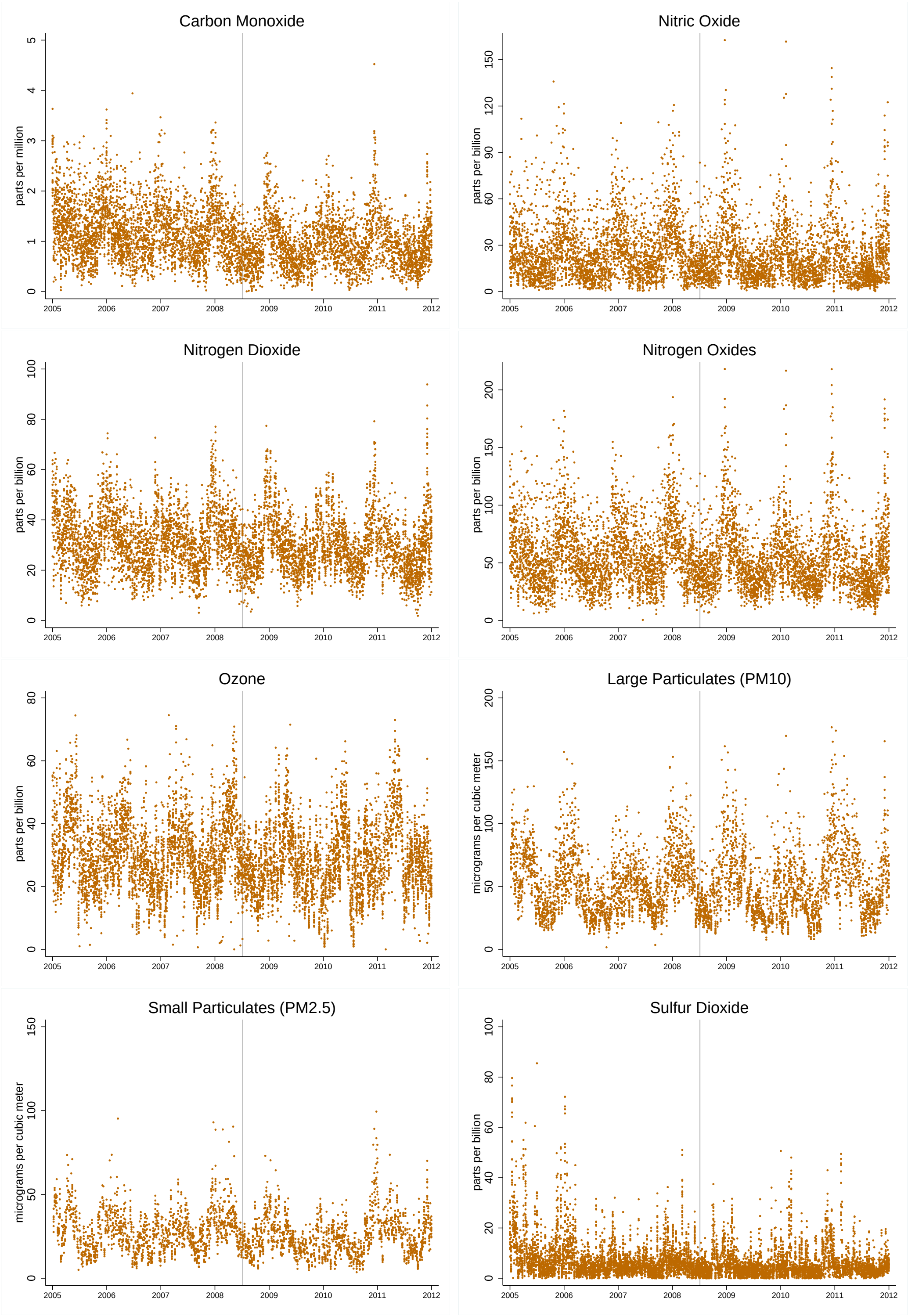

Appendix Figure 5: Weekends in Mexico City

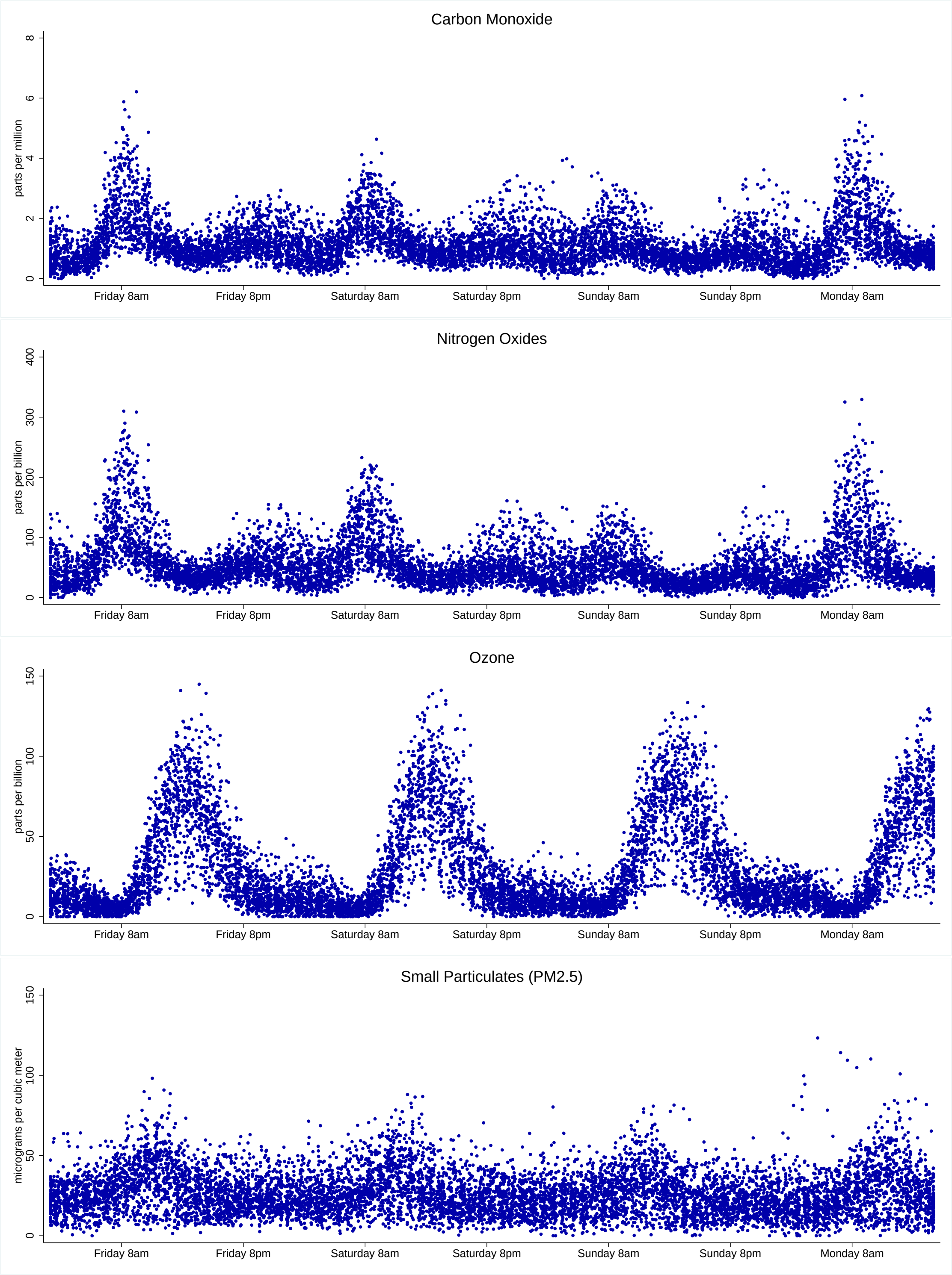

Appendix Figure 6: Weekends in Mexico City

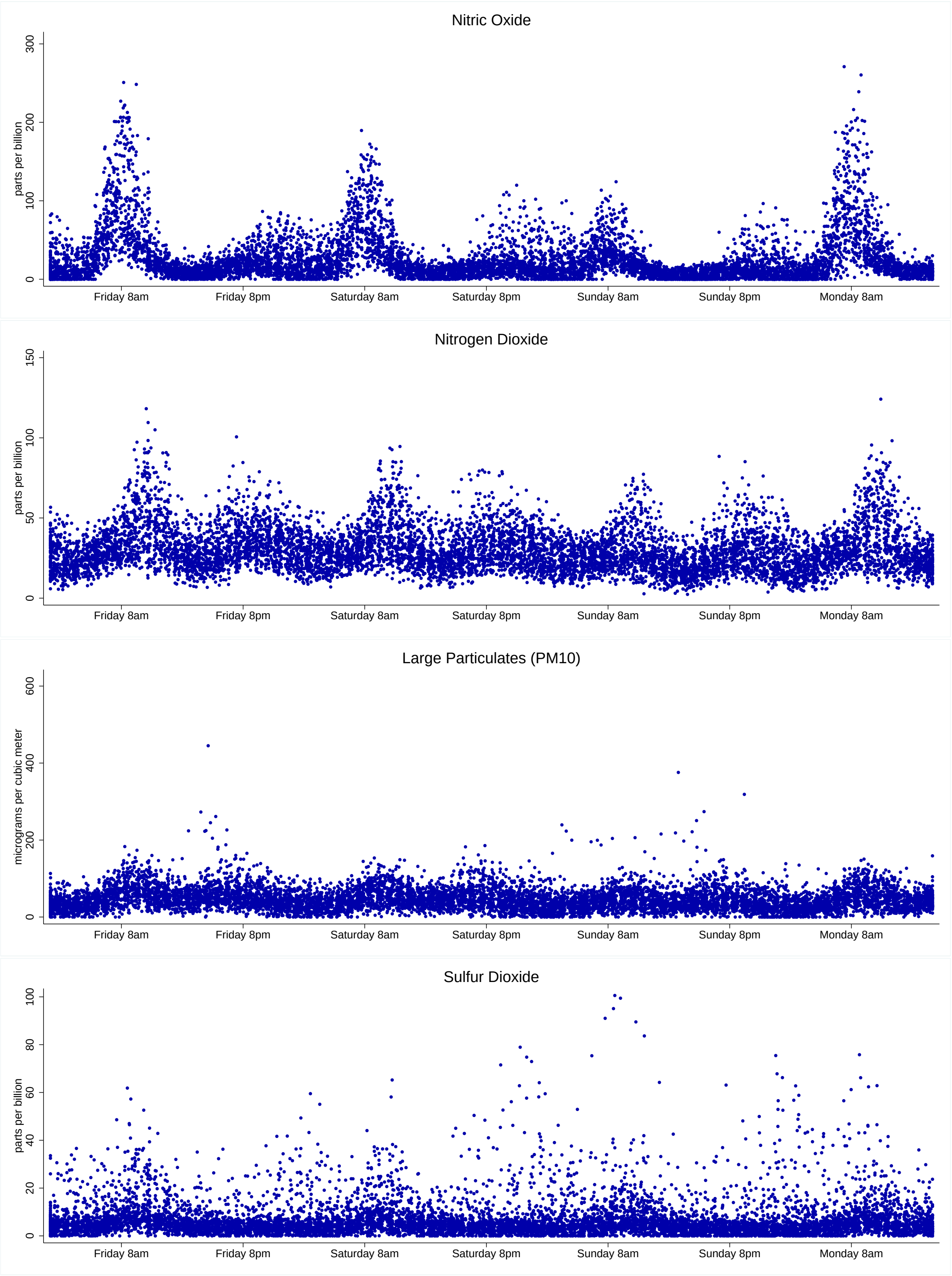

Appendix Figure 7: Maximum Daily Air Pollution on Saturdays in Mexico City

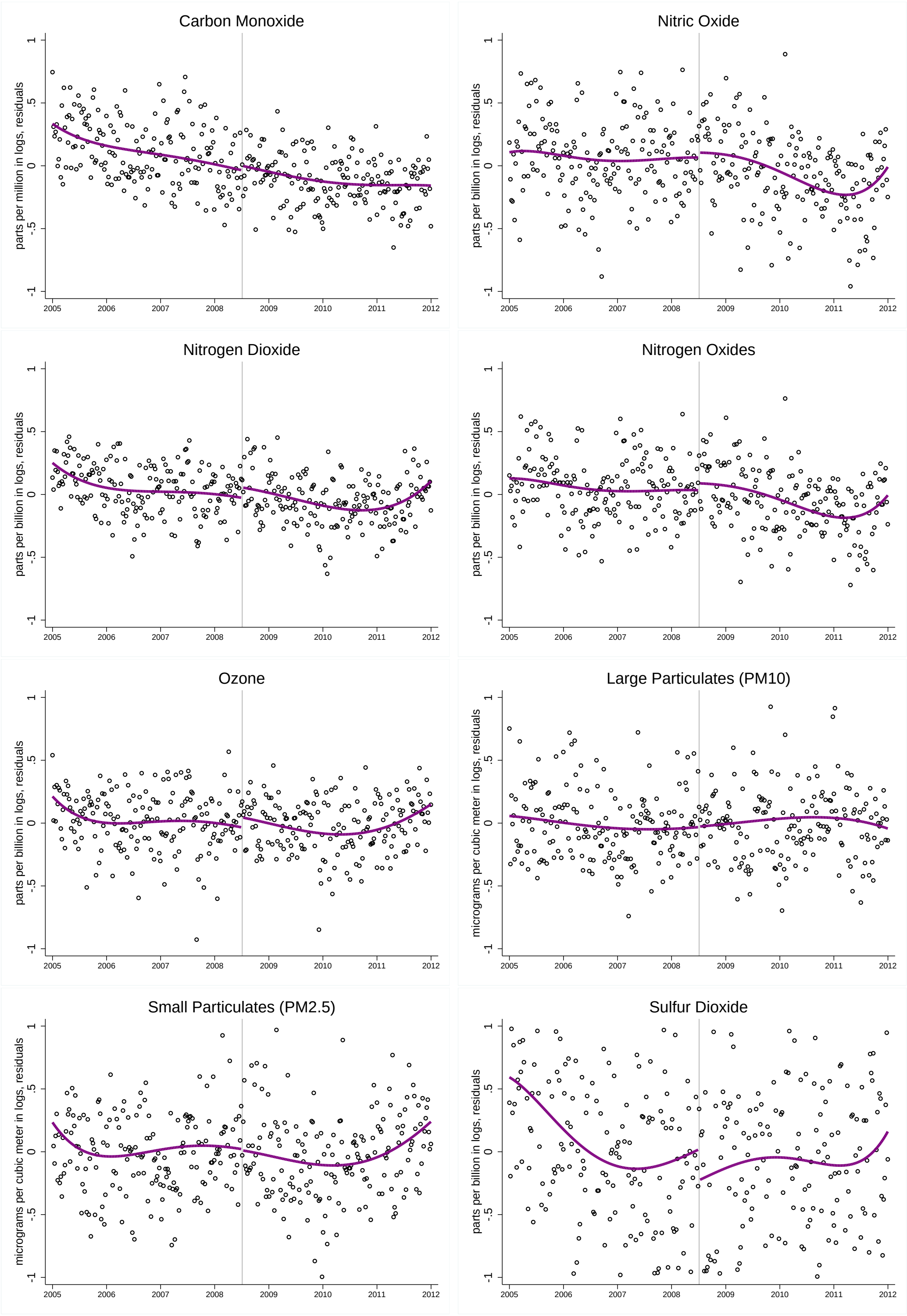

Appendix Figure 8: Regression Discontinuity Estimates by Hour

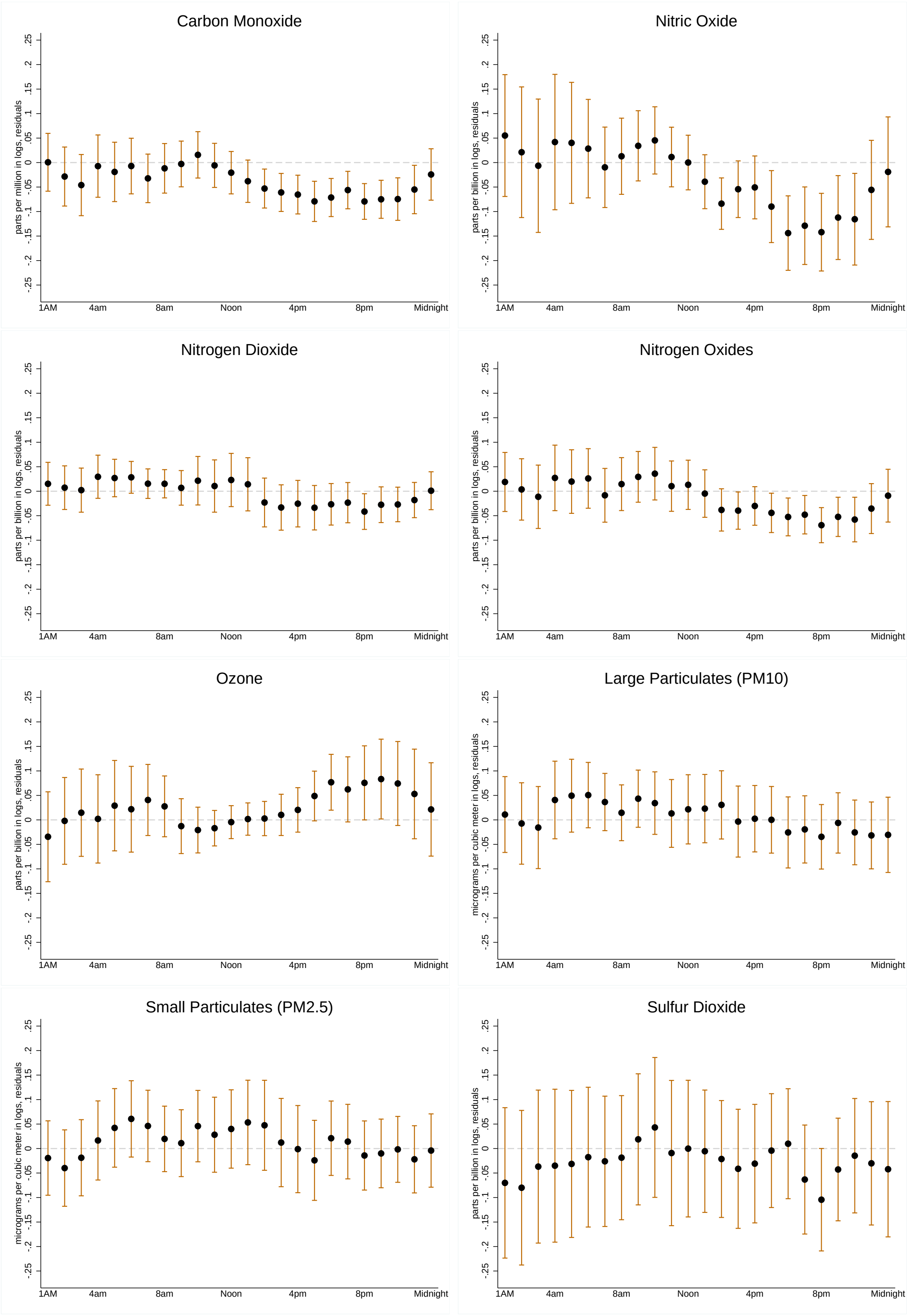

Appendix Figure 9: Mexico City Bus Rapid Transit Ridership, Raw Daily Data

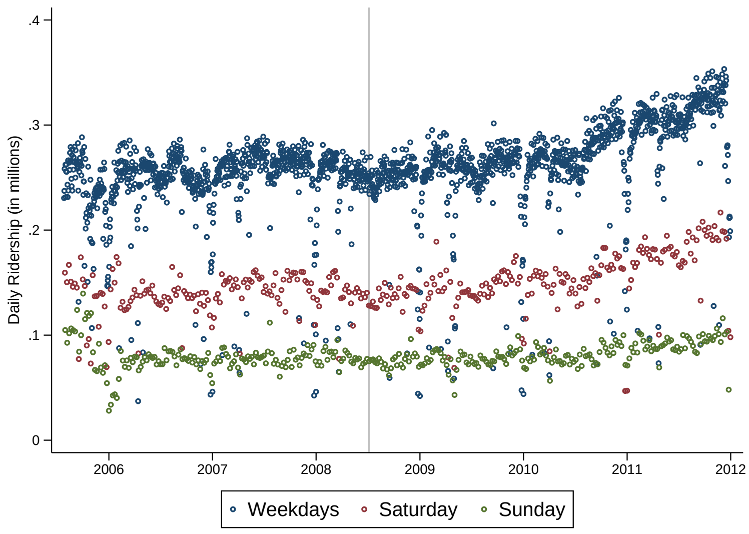

Source: *Gobierno de la Ciudad de México, Sistema Metrobus*, Freedom of Information Act Request, 2015.

Appendix Figure 10: Mexico City Light Rail Ridership, Raw Daily Data

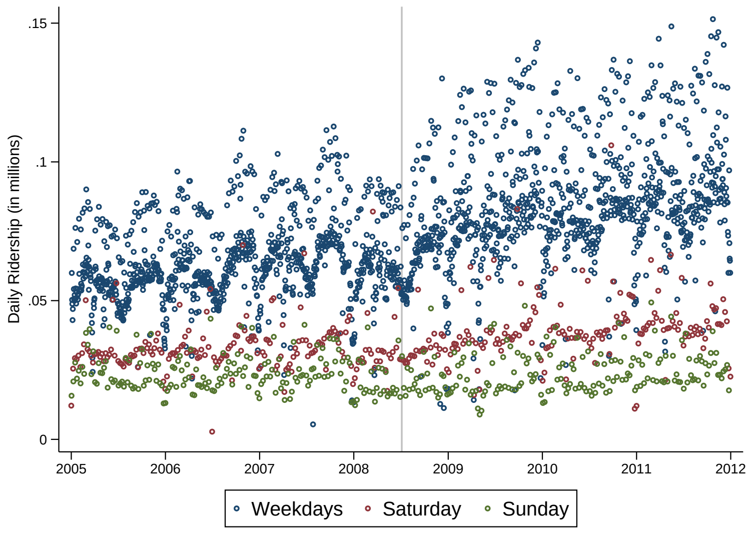

Source: *Servicio de Transportes Eléctricos del Distrito Federal*, Freedom of Information Act Request, 2015.

Appendix Figure 11: Mexico City Electric Bus System Ridership, Raw Daily Data

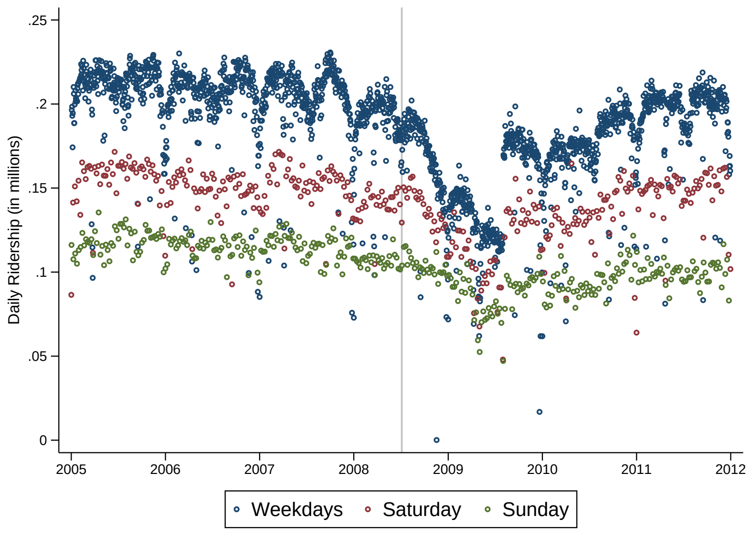

Source: *Servicio de Transportes Eléctricos del Distrito Federal*, Freedom of Information Act Request, 2015.

Appendix Figure 12: Mexico City Subway Ridership, Raw Daily Data

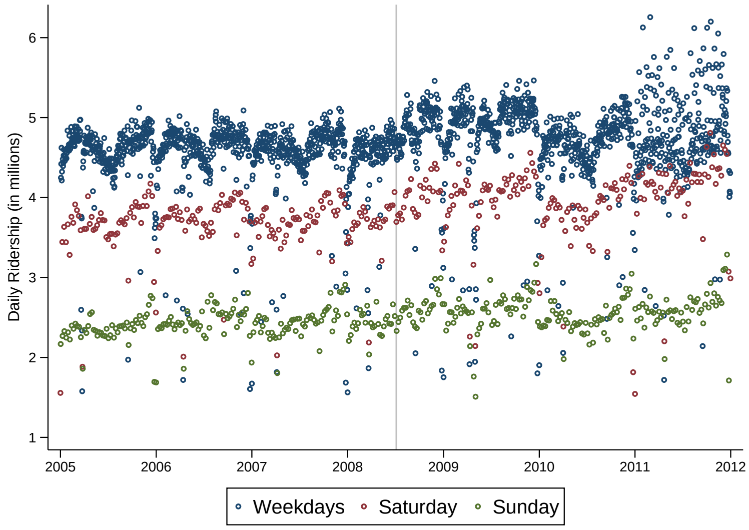

Source: *Gobierno de la Ciudad de México, Sistema de Transporte Colectivo Metro*, Freedom of Information Act Request, 2015.

Appendix Figure 13: Mexico City Zoo Admissions, Raw Daily Data

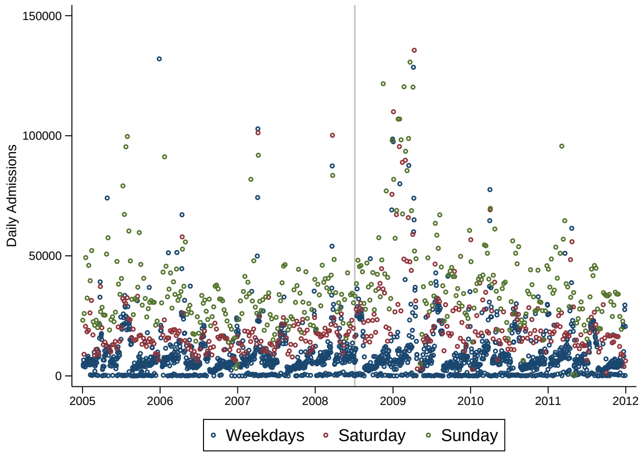

Source: *Zoológico de Chapultepec de la Ciudad de México*, Freedom of Information Act Request, 2015.

Appendix Figure 14: Mexico City Anthropology Museum Admissions, Raw Daily Data

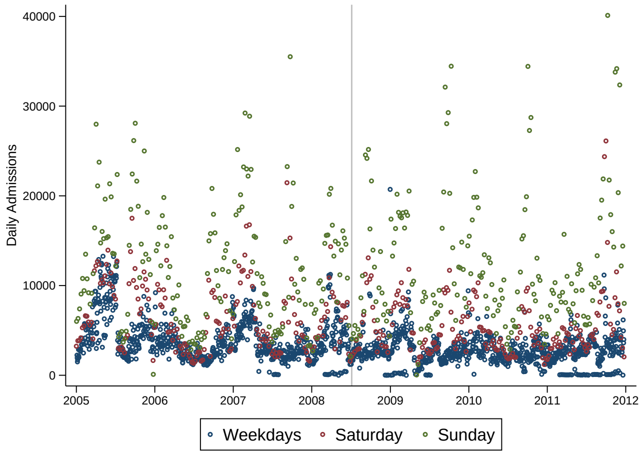

Source: *Museo Nacional de Antropología*, Freedom of Information Act Request, 2015.

Appendix Figure 15: Parking at Mexico City International Airport, Raw Daily Data

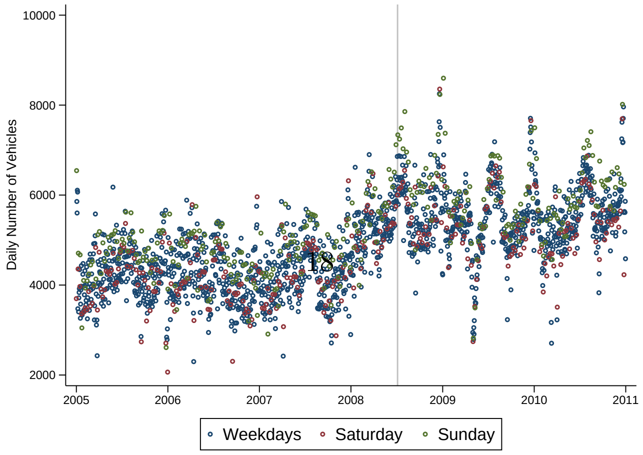

Source: *Aeropuerto Internacional de la Ciudad de México*, Freedom of Information Act Request, 2015.

Appendix Figure 16: Mexico City Bus Rapid Transit Ridership, Sunday to Friday

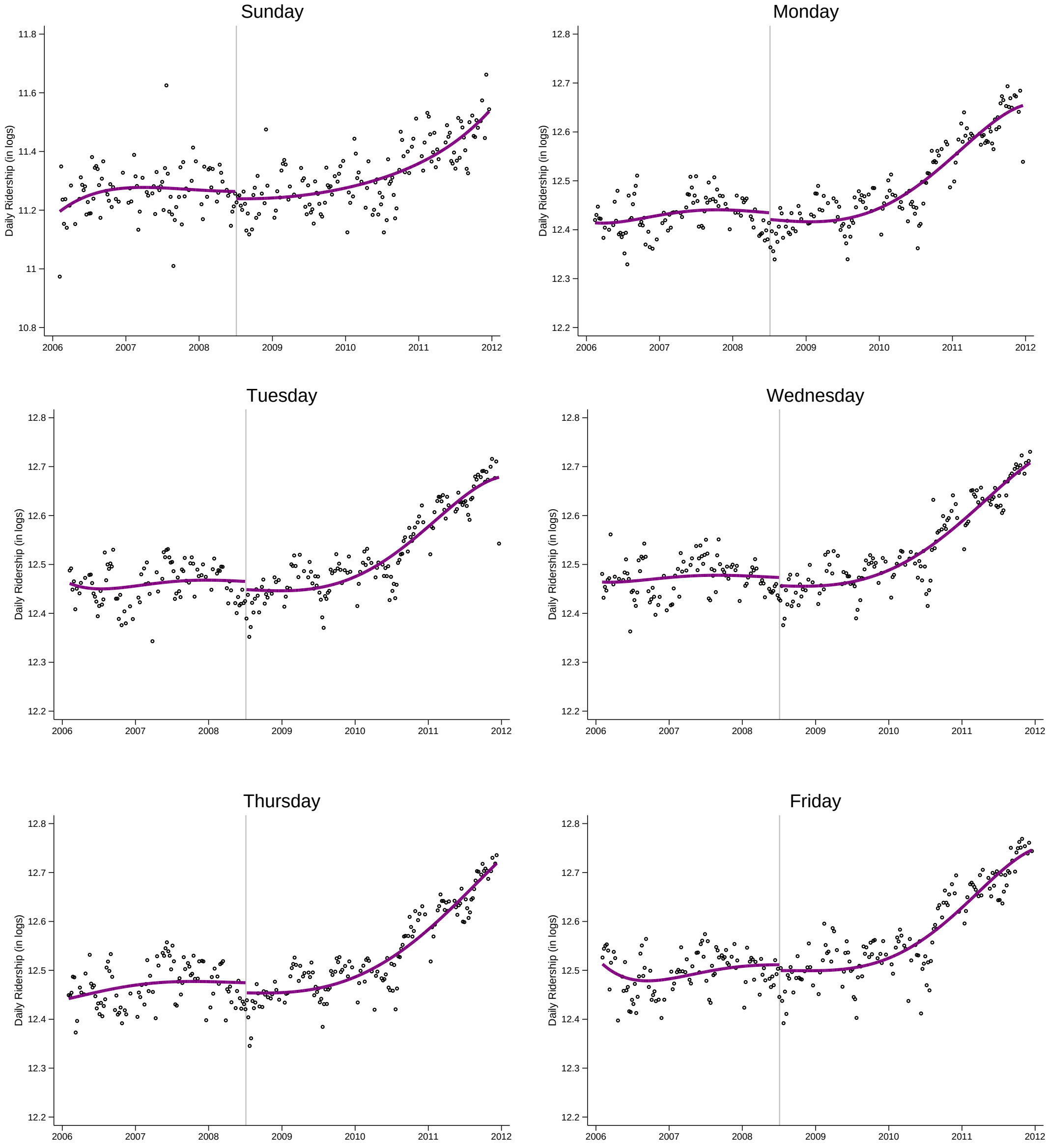

Appendix Figure 17: Mexico City Light Rail Ridership, Sunday to Friday

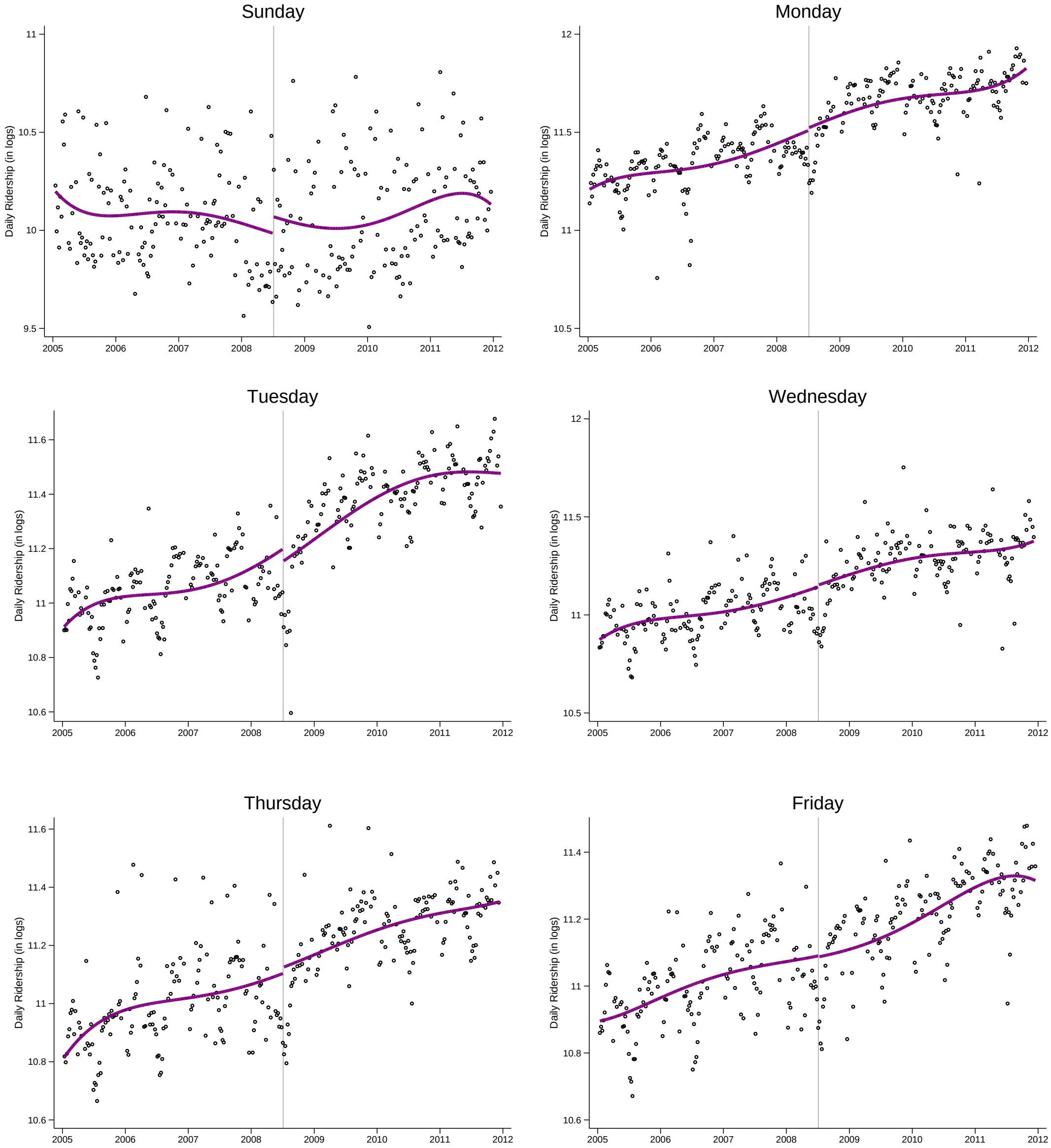

Appendix Figure 18: Mexico City Electric Bus System Ridership, Sunday to Friday

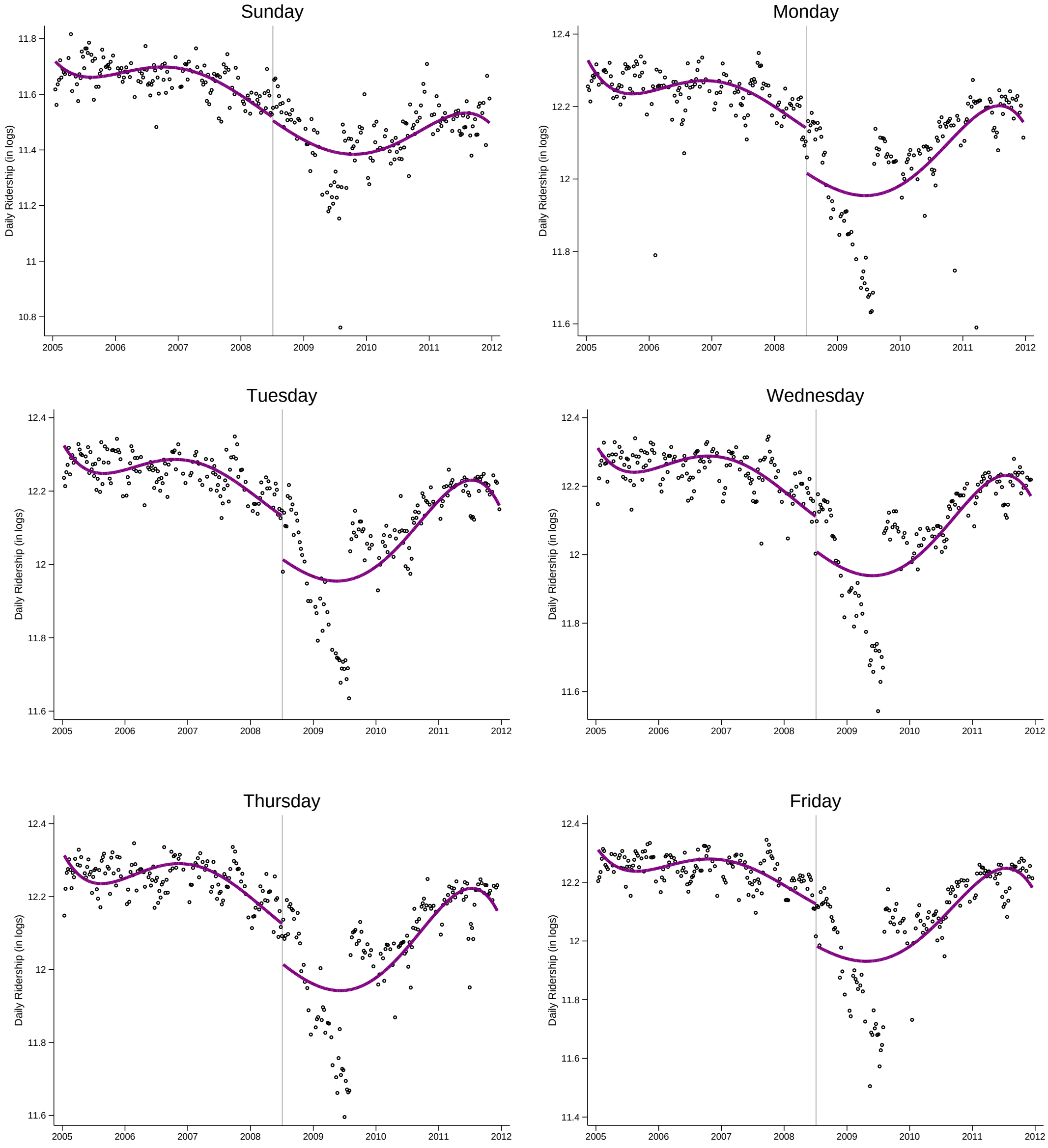

Appendix Figure 19: Mexico City Subway Ridership, Sunday to Saturday

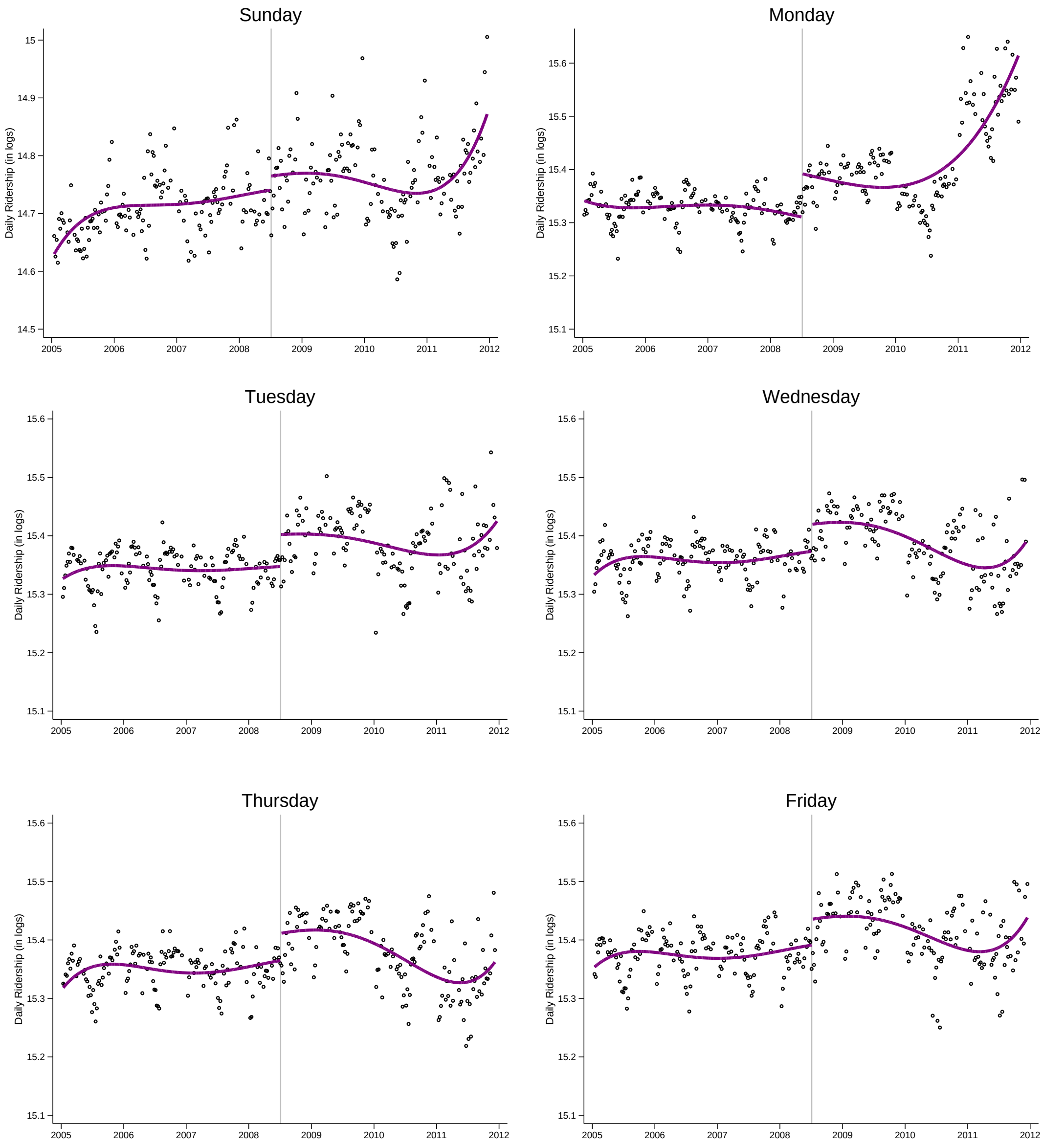

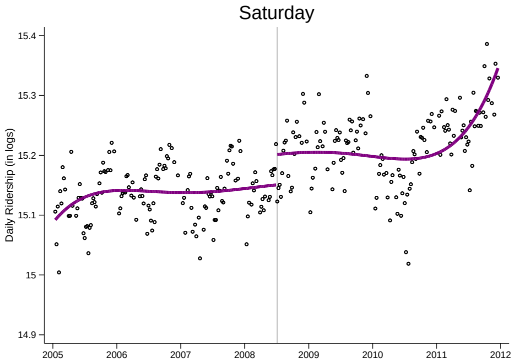

Appendix Figure 20: Mexico City Zoo Admissions, Sunday to Friday

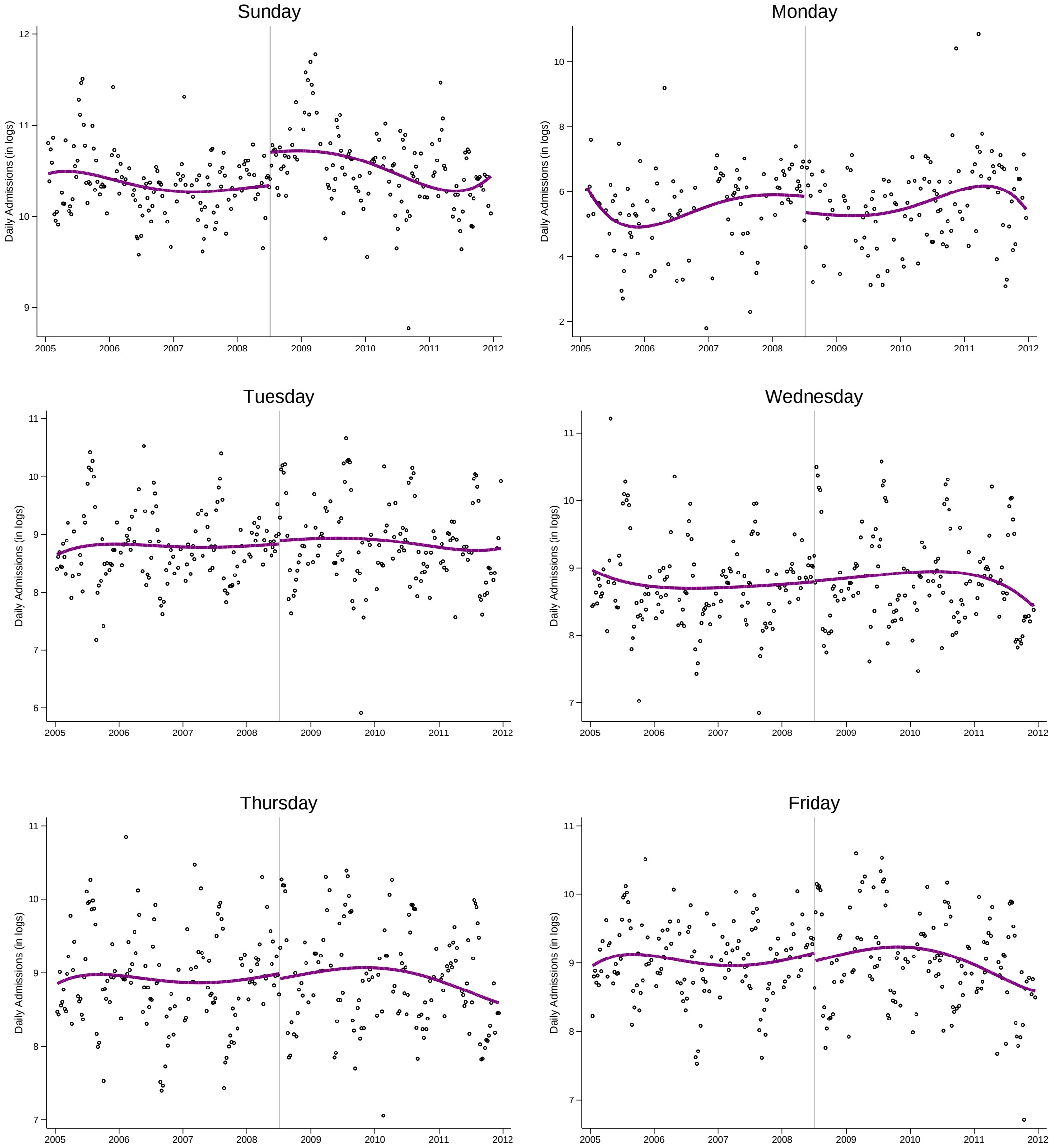

Appendix Figure 21: Mexico City Anthropology Museum Admissions, Sunday to Friday

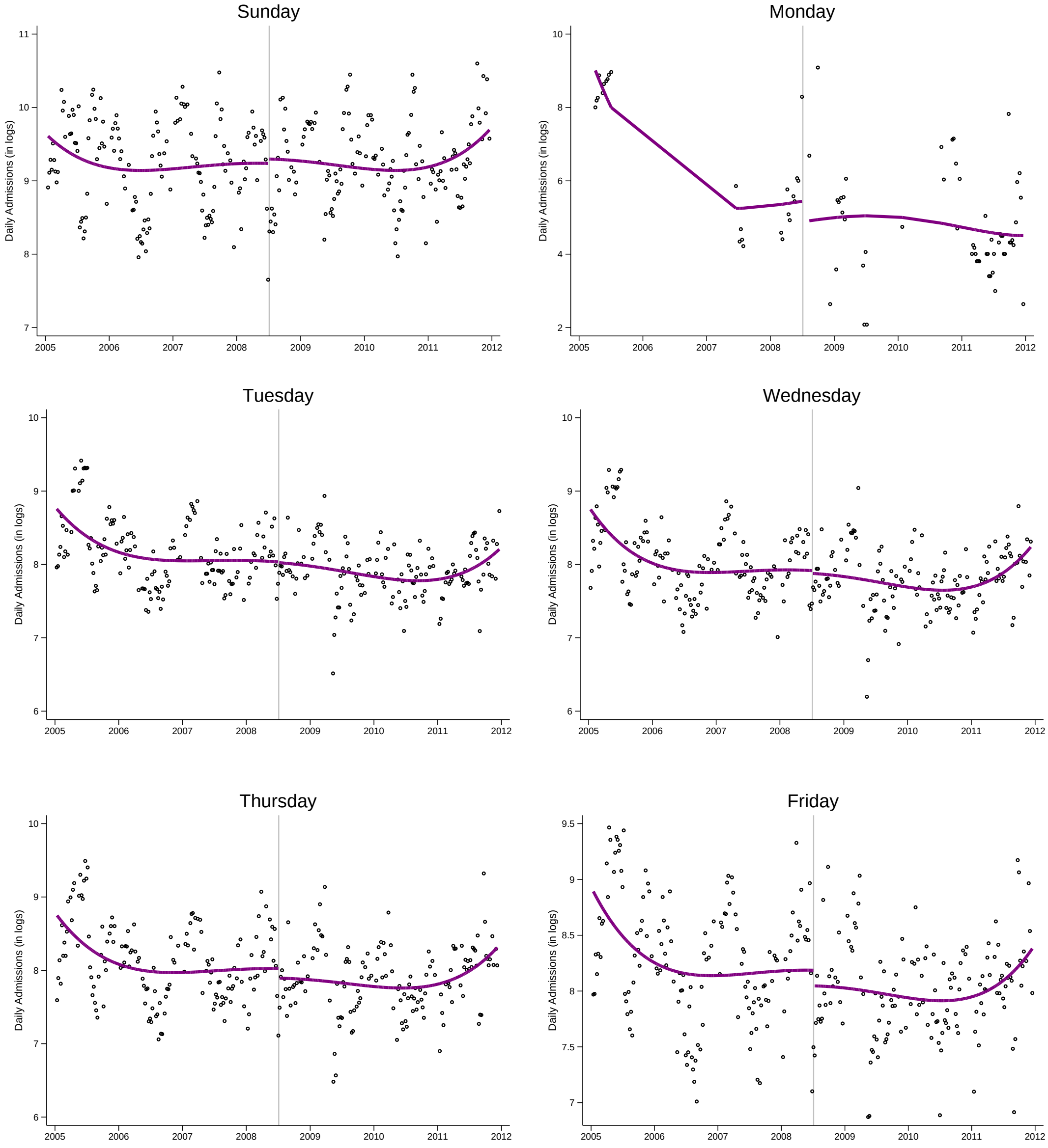

Appendix Figure 22: Parking at Mexico City International Airport, Sunday to Friday

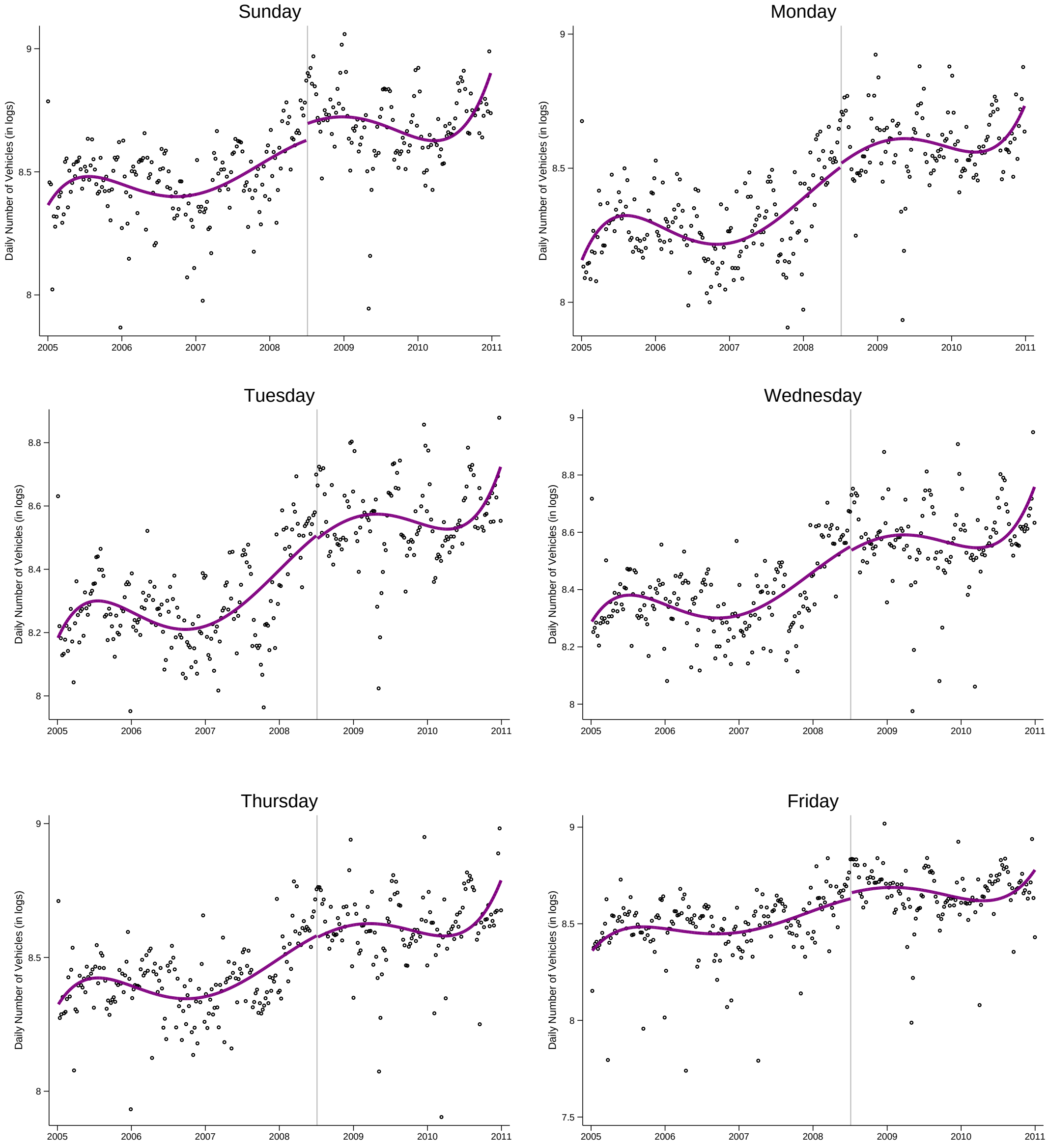

Appendix Table 1: Air Pollution in the Western Hemisphere's Ten Largest Cities

City	Large Particulates PM_{10}	Small Particulates $PM_{2.5}$	Urban Area Population, in millions
Mexico City, Mexico	93.4	24.8	20.1
Rio de Janeiro, Brazil	67.0	36.4	11.7
Lima, Peru	62.6	37.9	10.8
Bogotá, Colombia	47.6	26.6	9.0
São Paulo, Brazil	34.6	19.0	20.4
Los Angeles, USA	33.3	20.0	15.1
Buenos Aires, Argentina	30.3	16.4	14.1
New York, USA	23.2	13.9	20.6
Toronto, Canada	24.2	8.1	6.5
Chicago, USA	21.8	13.1	9.2

Note: Annual average airborne particulates in micrograms per cubic meter are from the World Health Organization's *Ambient Air Pollution in Cities Database 2014*.

Appendix Table 2: Descriptive Statistics

	(1) Observations	(2) Mean	(3) Std. Dev.	(4) Minimum	(5) Maximum
Carbon Monoxide (ppm)	697,764	1.048	0.869	0	11.70
Nitric Oxide (ppb)	704,977	25.95	40.73	0	702
Nitrogen Dioxide (ppb)	704,977	30.26	17.13	0	217
Nitrogen Oxides (ppb)	745,710	55.72	51.33	0	783
Ozone (ppb)	839,469	28.21	28.79	0	211
PM10 (ug/m3)	527,779	51.03	38.02	1	1,570
PM2.5 (ug/m3)	331,916	25.45	17.40	1	777
Sulfur Dioxide (ppb)	962,876	6.272	10.94	0	369
Temperature (Celsius)	477,774	16.40	5.220	-4.600	33.80
Humidity	483,667	52.82	22.27	1	100
Wind Speed (meters/second)	474,012	1.698	1.181	0.100	12.30

Note: These descriptive statistics were calculated by the author using data from Mexico City's *Red Automática de Monitoreo Atmosférico* (RAMA) and describe hourly pollutant and weather observations during the period 2006-2010 from all monitoring stations. The number of observations differs across variables because stations vary in what statistics are measured and because there are missing observations. Pollutants are measured in parts per million, parts per billion, or micrograms per cubic meter as indicated.

Appendix Table 3: Alternative Standard Errors

CO	NO	NO_2	NO_X	O_3	PM_{10}	$PM_{2.5}$	SO_2	Stacked
				Newey-West Standard Errors with 7-Day Lag				
-0.010	0.008	-0.001	0.011	0.024	0.027	0.011	-0.028	0.005
(0.028)	(0.013)	(0.016)	(0.021)	(0.021)	(0.027)	(0.056)	(0.015)	(0.010)

Note: These results are identical to the baseline estimates except that the coefficients and standard errors come from estimating a Newey-West specification with a 7-day lag.

Appendix Table 4: Alternative Polynomials

CO	NO	NO_2	NO_X	O_3	PM_{10}	$PM_{2.5}$	SO_2	Stacked
				Third-Order Polynomial				
-0.036*	-0.005	0.015	0.005	0.013	0.021	0.016	0.016	0.006
(0.014)	(0.027)	(0.012)	(0.016)	(0.020)	(0.020)	(0.026)	(0.054)	(0.016)
				Fourth-Order Polynomial				
-0.036*	-0.005	0.015	0.005	0.013	0.021	0.016	0.015	0.006
(0.014)	(0.027)	(0.012)	(0.016)	(0.020)	(0.020)	(0.026)	(0.054)	(0.016)
				Fifth-Order Polynomial (Baseline Estimates)				
-0.028*	-0.010	0.008	-0.001	0.011	0.024	0.027	0.011	0.005
(0.014)	(0.027)	(0.012)	(0.016)	(0.020)	(0.020)	(0.025)	(0.054)	(0.016)
				Sixth-Order Polynomial				
-0.028*	-0.010	0.008	-0.001	0.012	0.026	0.029	0.013	0.006
(0.014)	(0.027)	(0.012)	(0.016)	(0.020)	(0.020)	(0.025)	(0.054)	(0.016)
				Seventh-Order Polynomial				
-0.022	-0.001	0.008	0.003	0.006	0.026	0.026	0.013	0.007
(0.014)	(0.027)	(0.012)	(0.016)	(0.020)	(0.020)	(0.025)	(0.054)	(0.015)

Note: These results are identical to the baseline estimates except for the order of the polynomial time trend as indicated in the panel headings. An asterisk denotes statistical significance at the 5% level.

Appendix Table 5: Additional Specifications With Shorter Time Windows

CO	NO	NO_2	NO_X	O_3	PM_{10}	$PM_{2.5}$	SO_2	Stacked
Cubic Polynomial, 2006-2010								
-0.032	0.006	0.009	0.007	0.014	0.035	0.025	0.067	0.016
(0.017)	(0.033)	(0.014)	(0.019)	(0.025)	(0.024)	(0.030)	(0.064)	(0.019)
Quadratic Polynomial, 2007-2009								
-0.035	0.002	0.009	0.005	0.024	0.008	0.006	-0.016	-0.006
(0.023)	(0.041)	(0.020)	(0.024)	(0.030)	(0.031)	(0.038)	(0.079)	(0.016)
Quadratic Polynomial, 2007-2009, No Week-Of-Year Indicators								
-0.031	0.020	0.005	0.012	0.010	0.003	-0.005	-0.008	0.001
(0.025)	(0.044)	(0.021)	(0.026)	(0.032)	(0.033)	(0.040)	(0.081)	(0.017)
Quadratic Polynomial, 2008 Only, No Week-Of-Year Indicators								
-0.005	0.094	-0.017	0.032	0.006	-0.002	0.003	-0.060	0.001
(0.045)	(0.085)	(0.038)	(0.048)	(0.051)	(0.058)	(0.071)	(0.154)	(0.017)
Linear Polynomial, 2008 Only, No Week-Of-Year Indicators								
0.007	0.119	-0.014	0.046	-0.009	0.007	0.008	-0.055	0.001
(0.048)	(0.085)	(0.039)	(0.049)	(0.052)	(0.059)	(0.072)	(0.155)	(0.017)

Note: These results are identical to the baseline estimates except for the order of the polynomial time trend and sample period as indicated in the panel headings. In addition, the first two panels include week-of-year indicator variable but the last three panels do not. None of the estimates are statistically significant at the 5% level.

Appendix Table 6: Testing for Intertemporal Substitution

	CO	NO	NO_2	NO_X	O_3	PM_{10}	$PM_{2.5}$	SO_2	Stacked
Friday,	-0.004	-0.005	-0.002	-0.003	0.003	-0.011	-0.027	-0.034	-0.010
All Hours	(0.015)	(0.027)	(0.012)	(0.016)	(0.018)	(0.019)	(0.024)	(0.054)	(0.015)
Sunday,	0.013	0.002	0.014	0.019	0.034	-0.031	-0.012	0.012	0.006
All Hours	(0.016)	(0.031)	(0.013)	(0.017)	(0.018)	(0.023)	(0.027)	(0.055)	(0.016)

Note: This table reports coefficient estimates and standard errors from 9 separate regressions, one per column. The specifications are identical to the baseline specification in the paper except instead of an indicator for Saturdays after July 5th, 2008, two separate indicators are included for Fridays and Sundays after July 5th, 2008. None of the estimates are statistically significant at the 5% level.
